# Stage-specific transcriptomic analysis reveals insights into the development, reproduction and biological function of allergens in the European house dust mite *Dermatophagoides pteronyssinus*

**DOI:** 10.1186/s12864-025-11703-w

**Published:** 2025-05-26

**Authors:** José Cristian Vidal-Quist, Félix Ortego, Bart N. Lambrecht, Stephane Rombauts, Pedro Hernández-Crespo

**Affiliations:** 1 Departamento de Biotecnología, Entomología Aplicada a la Agricultura y la Salud, Centro de Investigaciones Biológicas Margarita Salas (CIB), CSIC, Madrid, Spain; 2 VIB-UGent Center for Inflammation Research, Ghent, Belgium; 3 Department of Internal Medicine and Pediatrics, Ghent University, Ghent, Belgium; 4 Center for Plant Systems Biology, VIB, Ghent, Belgium; 5 Department of Plant Biotechnology and Bioinformatics, Ghent University, Ghent, Belgium

**Keywords:** House dust mite, *Dermatophagoides pteronyssinus*, RNAseq, Allergen, Development, Reproduction, Seminal fluid protein, Ecdysteroid, Cuticular protein, Horizontal gene transfer

## Abstract

**Background:**

House dust mites (HDMs) such as *Dermatophagoides pteronyssinus* are major allergy elicitors worldwide, yet their gene expression across developmental stages remains underexplored. Herein, we report a comprehensive RNAseq analysis of larvae, nymphs, and adult males and females, mapped to a recently published high-quality genome with extended functional annotations.

**Results:**

Analysis of differentially expressed genes (DEG) revealed that female-biased expression was the most prevalent profile (16% of genes), while males exhibited the highest fold-change differences. DEG data, combined with network clustering and functional enrichment analysis, highlighted distinct genes and biological processes for each stage and sex: females showed upregulation of genes related to cell division and oogenesis, with vitellogenins among the most abundant transcripts; males exhibited increased expression of genes encoding putative seminal fluid proteins (e.g. endopeptidases, serpins, antimicrobial peptides), and those involved in reproductive regulation (e.g. testis-specific serine kinases); while juveniles displayed enhanced expression of genes related to energy metabolism and growth. Further analysis of endocrine pathways revealed non-canonic mechanisms compared to insect models, particularly in ecdysteroid and sesquiterpenoid biosynthesis and regulation. Expression patterns in genes involved in cuticle formation were also identified, reflecting their role in developmental transitions and sexual differentiation. Allergen and allergen-related gene expression showed an overall increase in feeding juveniles, as well as sex-biased expression, with Der p 27 upregulated in females. These findings provide insight into the physiological roles of allergens in digestion, immunity, and muscle formation, among other functions. Additionally, seven new horizontally transferred genes, including a DNA-repair photolyase linked to females, and novel multigene families (e.g. 119 male-specific beta-propeller proteins, 70 hypothetical cuticular proteins, 23 tetraspanin-like proteins, 5 female-associated putative odorant-binding proteins) were identified.

**Conclusions:**

This study provides the first genome-wide transcriptomic analysis of a HDM across life stages and sexes, expanding our understanding of the molecular mechanisms underlying mite development, sexual reproduction, and allergen expression. The generated data, fully available via supplementary spreadsheet and the ORCAE online platform, provide a valuable foundation for future allergy research and the development of new mite control strategies.

**Supplementary Information:**

The online version contains supplementary material available at 10.1186/s12864-025-11703-w.

## Background

House dust mites (HDMs) of the family Pyroglyphidae (suborder Astigmata, order Sarcoptiformes) are detritivorous species dwelling in close association with animal hosts, including humans (being found in bedding, furniture, carpets, etc., in homes) [1]. HDMs feed on shed skin and other organic detritus, which are colonized by molds and bacteria. Notably, HDMs are a global health problem because of their ability to elicit strong allergic reactions in susceptible humans and pets as a result of the production of various types of allergenic proteins (i.e. allergens). These mite-derived proteins are released into the environment via fecal particles or dead body fragments, which become airborne among other dust particles [2]. *Dermatophagoides pteronyssinus* (*Dp*), one of the most clinically relevant and widespread species, is known to express at least 34 different allergens [3]. The species particular physiology is key to understand allergenicity since some allergens have been linked to essential biological processes such as digestion (groups 1, 3, 4, 6, 9), cytoskeleton organization (groups 10, 11, 16, 26, 33, 39) or chitin metabolism and function (groups 15, 18, 23, 37), among others [1, 4, 5].

The *Dp* life cycle consists of 6 stages: egg, prelarva (still within the egg), larva, protonymph, tritonymph, and adult. Pyroglyphid species such as *Dp* are obligate sexual reproducers that exhibit sexual dimorphism: adult males are smaller than females, are more sclerotized, and have distinctive structures like the anal suckers, which allow grasping to the female during copulation [1]. The active feeding larval and nymphal stages are followed by quiescent periods prior to molting (pharate stages). The pharate tritonymph, in particular, is believed to be the quiescent stage that allows survival under suboptimal environmental conditions (e.g. at low relative humidity and temperature during winter) and the subsequent recovery of mite colonies [6]. As a result, the age/stage structure of mite populations in natural habitats and homes tends to show seasonal variations. In addition, despite typically having a 1:1 sex ratio, adult sex proportions in *Dermatophagoides* populations are also susceptible to shifts caused by a number of factors, as observed in laboratory cultures [7]. Notably, population structure dynamics might be relevant to allergy since it could directly impact the allergenic profile of mite culture extracts used in the manufacture of pharmaceutical products [8]. To date, our knowledge of the expression of *Dp* allergens at different developmental stages is still limited.

The physiology of mites is generally poorly characterized compared with that of insects. Despite considerable efforts in understanding allergen biochemistry and molecular biology in HDM, this knowledge gap is illustrated by the lack of functional information on many of the described allergens, including clinically important allergens such as group 2 major allergens. Mite-derived transcriptomic resources can provide valuable information on gene function and fundamental biological processes such as development or reproduction, as well as help identify genes and proteins of biotechnological interest. Examples of the latter include enzymes suitable for the biomass/biowaste valorization industry like chitinolytic enzymes [9, 10], or targets for the development of new active ingredients in the control of economically and medically important mites [11, 12]. Chemical control of HDMs using conventional synthetic acaricides and natural products has been proposed and, to some extent, applied in the past [1, 13]; however, this control measure is generally not recommended in homes because of safety and/or efficacy concerns [2, 14]. Novel approaches exploiting genomic information for the rational design of pesticides are currently emerging which may provide unprecedented opportunities for HDM and mite control in general. These include the discovery of new active molecules by combining computational methods for molecular docking, virtual screening of compound libraries and protein structure modeling [15–17]; or the application of exogenous acaricidal dsRNA against target-specific essential genes via spray-induced gene silencing. The potential of RNAi as a technique to alter arthropod physiology has long been validated, including in HDMs [18, 19], but only recently have the first commercial RNAi-based pesticides come to market owing to advancements in the large-scale dsRNA synthesis and optimized formulation/delivery methods [20, 21].

A number of genomic assemblies are currently available for *Dp* and other HDMs [22–24]. Our group recently released a high-quality annotated genome assembly obtained from a *Dp* inbred laboratory colony [25, 26]. The annotation of this assembly is gradually being improved by manual curation and is publicly available at the Online Resource for Community Annotation of Eukaryotes (ORCAE) platform [27, 28]. As a result of these genomic data, whole-genome transcriptomic studies have been recently reported allowing new insights into the physiology of this key HDM species [25, 29]. These resources, together with the species small size, short generation time, simple rearing and already optimized bioassay methods [5, 19, 30, 31], make *Dp* a good candidate as a model for studying other Astigmata mites. This suborder includes other allergy-eliciting HDMs, pests of stored food products and parasitic mites of medical and veterinary importance [1, 24]. Analyzing development-specific transcriptomes is a fundamental tool for studying arthropod biology, which has already been addressed in a number of mite and tick species [32–37]. However, except for a single RT-qPCR-based study focused on a limited number of *Dp* allergen genes [8], this type of transcriptomic information is currently unavailable for HDMs. Hence, this study represents the first whole-transcriptome evaluation of gene expression across developmental stages and adult sexes of *Dp*, or other HDMs, and provides insight into their biology, with a particular focus on reproduction, development and allergen expression and function. The generated transcriptomic data complement previous -omic resources available on *Dp* and support future research on this clinically important species, other HDM, and Astigmata mites in general.

## Methods

### Mite cultures and sampling

The maintenance of our *Dp* stock colony was conducted as previously described [8]. Mites of specific developmental stages (larvae or nymphs; the latter including both protonymphs and tritonymphs) or adult sexes (males and females) were separated from stock cultures under a stereomicroscope according to their morphological traits [1]. Crawling mites deprived of media/fecal particles and placed onto the surface of a paper disk were collected using a vacuum pump connected to a sterile microtip and immediately submerged in ice-chilled TRIzol Reagent (Life Technologies, Carlsbad, USA), as described elsewhere [8]. Samples of 200–1500 individual mites (depending on the stage) were homogenized using a microtube pellet pestle and stored at -80 °C.

### RNA sequencing and analysis of gene expression

Total RNA was extracted from TRIzol homogenates following the manufacturer´s instructions and using glycogen as a carrier. The RNA was diluted in RNAsecure Resuspension Solution (Life Technologies). RNA quantity and quality were assessed via microvolume spectrophotometry followed by microfluidic chip electrophoresis using the Experion Automated Electrophoresis System (Bio-Rad, Hercules, USA). For RNA sequencing (RNAseq), 12 Illumina strand-specific total RNA paired-end libraries (3 replicates per stage/sex) were run on an Illumina HiSeq2500 150-bp paired-end sequencing system in HiSeq V4 high output mode (NCBI Bioproject ID PRJNA1233935). Bioinformatic analysis of sequencing reads was conducted with the tools available at the Galaxy Europe server [38, 39], with our *Dp* genome assembly (NCBI GCA_027571235.1; Bioproject ID PRJNA843460) and annotation available at the ORCAE platform used as a reference [28]. Differential expression analysis was performed for each pairwise comparison of stages/sexes according to the following pipeline: Cutadapt > HISAT2 > StringTie (using annotated transcripts as reference; GTF3 format) > DESeq2. The frequencies of differentially expressed gene (DEG) profiles were compared by Venn diagram analysis using InteractiVenn [40].

Basal transcription levels were estimated via the transcripts per million (TPM) metric computed using StringTie, which normalizes gene expression count data. Principal component analysis (PCA) was performed with the *prcomp* function in R under the program RStudio (version 2024.04.2), with scaling applied to center the data and ensure equal weights for all genes. The PCA plot was visualized using *ggplot2* in R. Heatmap analyses were generated using the R package *pheatmap* (version 1.0.12) with a Euclidean clustering method. Whole-transcriptome gene–gene network clustering analysis was conducted within the Graphia v4.0 package, using stage-specific TPM values as input and a Markov cluster algorithm method [41].

Functional enrichment analysis based on Gene Ontology (GO) terms was conducted using GOEnrichment (Galaxy Version 2.0.1) [42] with the Core Gene Ontology file go.obo (2023–04-01). KEGG (Kyoto Encyclopedia of Genes and Genomes) pathway enrichment analysis was conducted with three different tools: ShinyGO, g:profiler and Metascape [43–45], using the FlyBase IDs (FBgn number; [46]) of *Drosophila melanogaster* proteins showing homology to the *Dp* genes under study, as previously described [29]. Enriched KEGG pathways were determined to be those showing significant enrichment (FDR < 0.05) with at least two of the bioinformatic tools. Visualization of genes showing different stage-related expression patterns against reference KEGG pathway maps was achieved by mapping previously annotated KEGG ontology (KO) terms [29] via the KEGG Mapper Colors tool, which is available at the KEGG website [47, 48].

### Annotation and classification of genes of interest

A number of gene annotation categories presented as supplementary data have already been reported in recent works by the group [25, 29]. These include protein domains and motifs; GO terms; KO terms; OrthoMCL orthology groups; *D. melanogaster* homologs; peptidases; peptidase inhibitors; lipases/esterases; glycosylases; immunity-related genes; cell adhesion/epithelial barrier genes; and allergy-related proteins.

For the new categories addressed in this report, genes were manually assigned to each category after following one or more of these methods: screening of relevant GO terms and InterProScan protein domains; BLASTp searches against NCBI databases or our *Dp* proteome; or crosslinking of annotated KO terms with relevant KEGG BRITE Hierarchy File and Pathway references using the KEGG Mapper Reconstruction tool [47, 48]. Putative cuticular protein-encoding and horizontal gene transfer (HGT) genes were identified applying additional methods, as described below.

### Screening of cuticular proteins (CPs)

#### First strategy: screening of CPs from known families

Common motifs previously identified in different CP families (CPR, CPF, TWDL, CPLCG, CPLCW, CPLCA, CPLCP, CPCFC, and CPAP) [49], as well as selected CP amino acid sequences from chelicerate species available at GenBank or obtained from the CuticleDB database [50] were used as BLASTp queries against the *Dp* genome using the ORCAE platform. Additionally, CP candidates were screened on the basis of InterPro protein domain annotations commonly found in CP: a) “insect cuticle protein” domain IPR000618, associated with the CPR family; and, b) “chitin-binding peritrophin A domain” (CBD, PFAM PF01607), integrated into the IPR036508 and IPR002557 domains, and associated with the CPAP family. The selected candidates were further classified using the CutProtFam-Pred webserver [51, 52].

#### Second strategy: screening of potentially undescribed CP families in Dp

Our annotated proteome was first filtered for proteins lacking InterPro and Panther domains (references with an IPR or PTHR prefix, respectively), GO term annotations, KO terms, or other inhouse annotations, such as inclusion in peptidase inhibitor or antimicrobial peptide (immunity) categories. Next, genes with high transcription level were selected on the basis of average TPM values from 4 RNAseq libraries of *Dp* control cultures, as previously reported [29]. A cutoff of 123.7 TPM units was selected to include genes within the first decile of expression for the whole transcriptome. The protein sequences were subsequently analyzed with SignalP 6.0 [53] to select proteins bearing a signal peptide and DeepLoc 2.0 [54] to select those with a predicted extracellular localization. Finally, a number of sequence/structural traits attributed to CPs in the literature [49, 55, 56] were inspected, including richness in hydrophobic amino acids such as glycine, proline, or alanine; lack of cysteine residues in the mature protein; and the presence of particular sequence attributes such as bearing three or more AAP(AVL) motifs, glycine dense regions, abundant PV and PY pairs, or tandem sequence repeats.

### Genomic screening of candidate horizontal gene transfer (HGT) events

The mining of our annotated *Dp* proteome for HGT candidates was conducted by adapting previously described methods [57]. Custom databases were built by retrieving available protein sequences from the RefSeq NCBI Reference Sequence Database [58] for a number of selected taxonomic phyla: invertebrate metazoan species excluding Acari (mites and ticks); fungi; bacteria; Archaea; Protozoa; and viruses. After a local BLASTp search of the *Dp* proteins against the different databases, the *h*-index was calculated for each of the selected non-metazoan phyla by subtracting the bitscore of the best non-Acari invertebrate alignment from the bitscore of the best non-invertebrate alignment (fungi; bacteria; Archaea; Protozoa; or viruses). Consequently, the higher the *h*-index is, the closer the target *Dp* sequence is to a potential non-metazoan donor clade. For the newly classified HGT genes in this study, HGT candidates were selected when the *h*-index was > 30 and the bitscore of the best non-metazoan alignment (potential donor) was > 100. It is to be noted that, by excluding Acari sequences from our metazoan invertebrate database, our strategy aimed to detect HGT events occurring only after the speciation events leading to the Acari lineage within Chelicerata. Consequently, other HGT genes additionally identified in this work but derived from previous studies may not meet the selection criteria described above, as these genes were deduced via potentially different methods (e.g. other genomic assemblies, protein databases and/or selection cutoffs).

### Protein structure prediction

Protein prediction models were generated using Alphafold 2 within the Galaxy platform (Galaxy Version 2.3.1 + galaxy4) [17] with default settings and were visualized via UCSF Chimera v1.17.3 (University of California). Structural homology to Protein Data Bank (PDB) crystallographic structures was assessed using the Phyre2 [59] and the PDBeFold [60] servers.

## Results and discussion

### General expression profiles across *D. pteronyssinus* stages

**Fig. 1:**
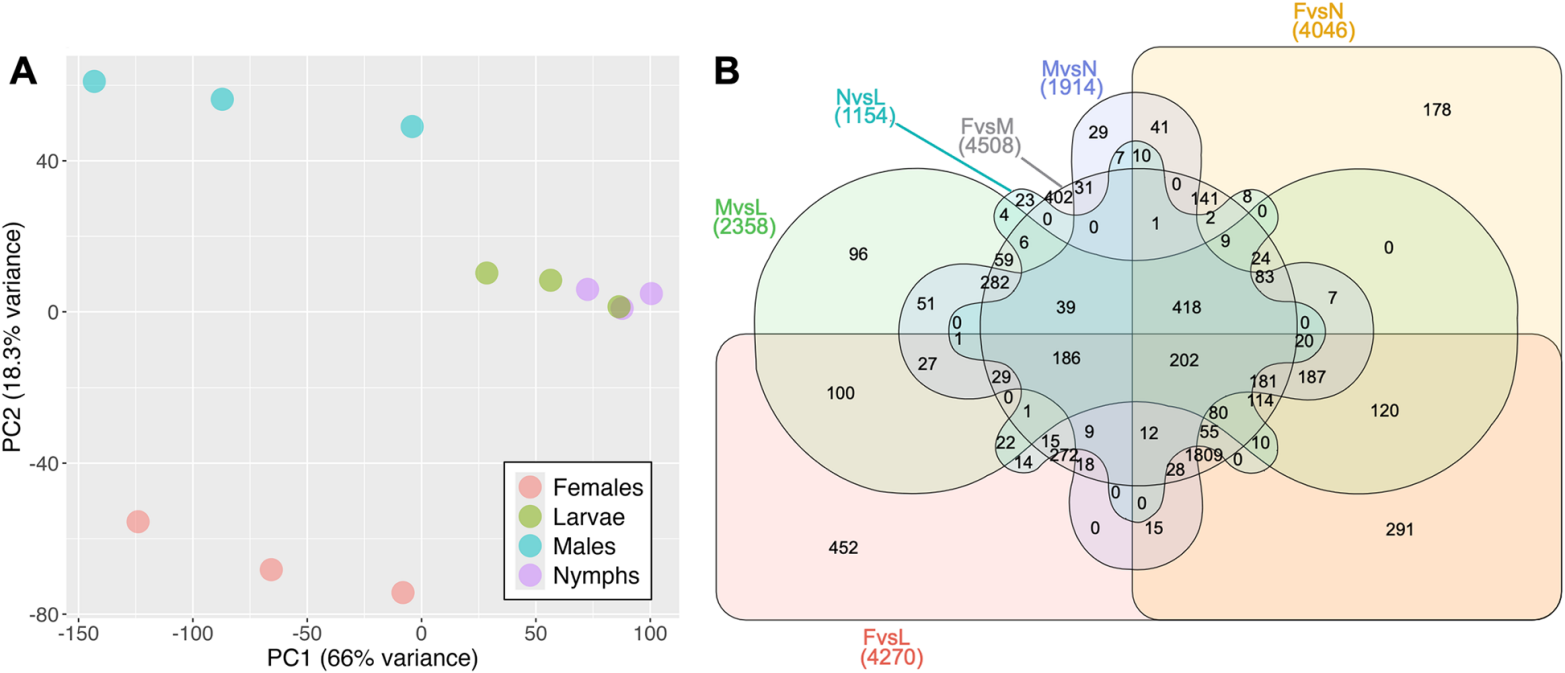
Whole transcriptome expression analysis comparing *Dermatophagoides pteronyssinus* developmental stages. Panel A) Principal component analysis plot of RNAseq gene expression data (transcript per million values) from the complete transcriptome across samples of four developmental stages. The plot displays the distribution of samples in the space of the first two principal component dimensions (PC1 and PC2), which explain 66% and 18.3% of the variance, respectively. Each point represents a biological replicate, colored according to the developmental stage (larvae, nymphs, females, males). The proximity of the points indicates the similarity in the gene expression profiles of their corresponding samples. Panel B) Six-way Venn diagram of the differentially expressed genes (DEGs) identified for each of the six pairwise comparisons between developmental stages*.* Each pairwise comparison is identified with a “XvsY” code, where “X” and “Y” denote the developmental stages under comparison (L: larvae; N: nymphs; F: females, M: males); numbers in brackets indicate the total number of DEGs for each comparison. Each of the six areas represents one comparison; intersections between areas highlight groups of genes showing the same expression profile (i.e. being differentially expressed under all the overlapped comparisons at the same time). The number of genes included in each group are depicted within each area or intersection

Illumina sequencing of the 12 analyzed libraries (3 biological replicates for each of the 4 collected stages; larvae, nymphs, adult females and males) produced an average of 22.3 to 25.1 million raw sequence reads per library (Additional file 1: Table S1). The average percentage of reads from each library aligning to our annotated genome assembly (NCBI GCA_027571235.1) ranged from 51.3% to 76.1%. This moderately low alignment rate is explained by the high abundance of raw reads derived from RNA viruses which, as shown previously, infect the stock colony used in this study and laboratory populations from different origins [61]. Principal component analysis comparing expression estimates (transcripts per million; TPM) for the 11,347 annotated genes across the different libraries revealed a clear demarcation between the different sampled stages and their corresponding replicates, with nymphs and larvae showing close proximity and females and males showing independent clusters (Fig. 1A).

Pairwise differential gene expression bioinformatic analysis between individual stages was conducted considering a false discovery rate (FDR)-adjusted *p* value ≤ 0.05 and a fold change cutoff ≥  ± 2. A total of 6221 genes (55% of the annotated genes) presented significant transcription differences in at least one pairwise comparison between stages following these criteria. For a number of selected genes, RNAseq-derived expression data were additionally confirmed by RT-qPCR, showing a generally high correspondence between both methods (Additional file 1: Tables S2 and S3). The frequencies of differentially expressed genes (DEGs) estimated by RNAseq for all six pairwise comparisons and their intersections (51 different profiles recorded out of 63 possible combinations) were revealed by Venn diagram analysis (Fig. 1B). The most common profile among DEGs corresponded to differences between females and all remaining libraries (*n* = 1809), whereas the number of genes significantly different between males and the rest of libraries was *n* = 282. Despite the lower frequency of male DEGs compared to female DEGs, the highest overexpression levels (fold-change) recorded for males were considerably greater than those recorded for females, suggesting that sex-specific gene expression is more noticeable in males. In comparison, a total of 466 genes exhibited > 50-fold overexpression in males compared to females, whereas no gene was overexpressed at this level in the opposite comparison.

For application throughout the following sections, any reference to a DEG showing differential expression in a particular stage/adult sex implies that all the corresponding pairwise comparisons are significantly different, unless indicated otherwise. Additionally, in this report, transcription profiles were studied via two other methods: network graph analysis, which allows genome-wide clustering of genes to identify biological processes and pathways linked to the life-cycle [34, 35] (see next section), and heatmap analysis, which was used to analyze transcription patterns within selected gene families/groups (as indicated in the text). Of note, both methods account for average TPM differences and can highlight DEGs that do not necessarily reach the fold-change cutoff described above.

Detailed transcriptomic information for each gene is available in Additional file 2, a spreadsheet where stage-specific expression profiles, annotation data (e.g. protein domains, GO terms, KEGG ontology, etc.), and assignments to a number of functional gene categories established in this and previous studies [25, 29] are displayed as separate columns. See the legend of Additional file 2 for more details on how to navigate through it. The resulting datasheet includes most of the results presented throughout the report for each of the studied categories and has been configured to be conveniently sorted and filtered by the user. Locus IDs in this table and throughout the text refer to the references available at ORCAE [28]. Thus, Additional file 2 aims at complementing the extensive information already available at the cited webserver, which has been updated along with this publication.

### Network analysis and functional annotation of stage-specific gene groups

**Fig. 2:**
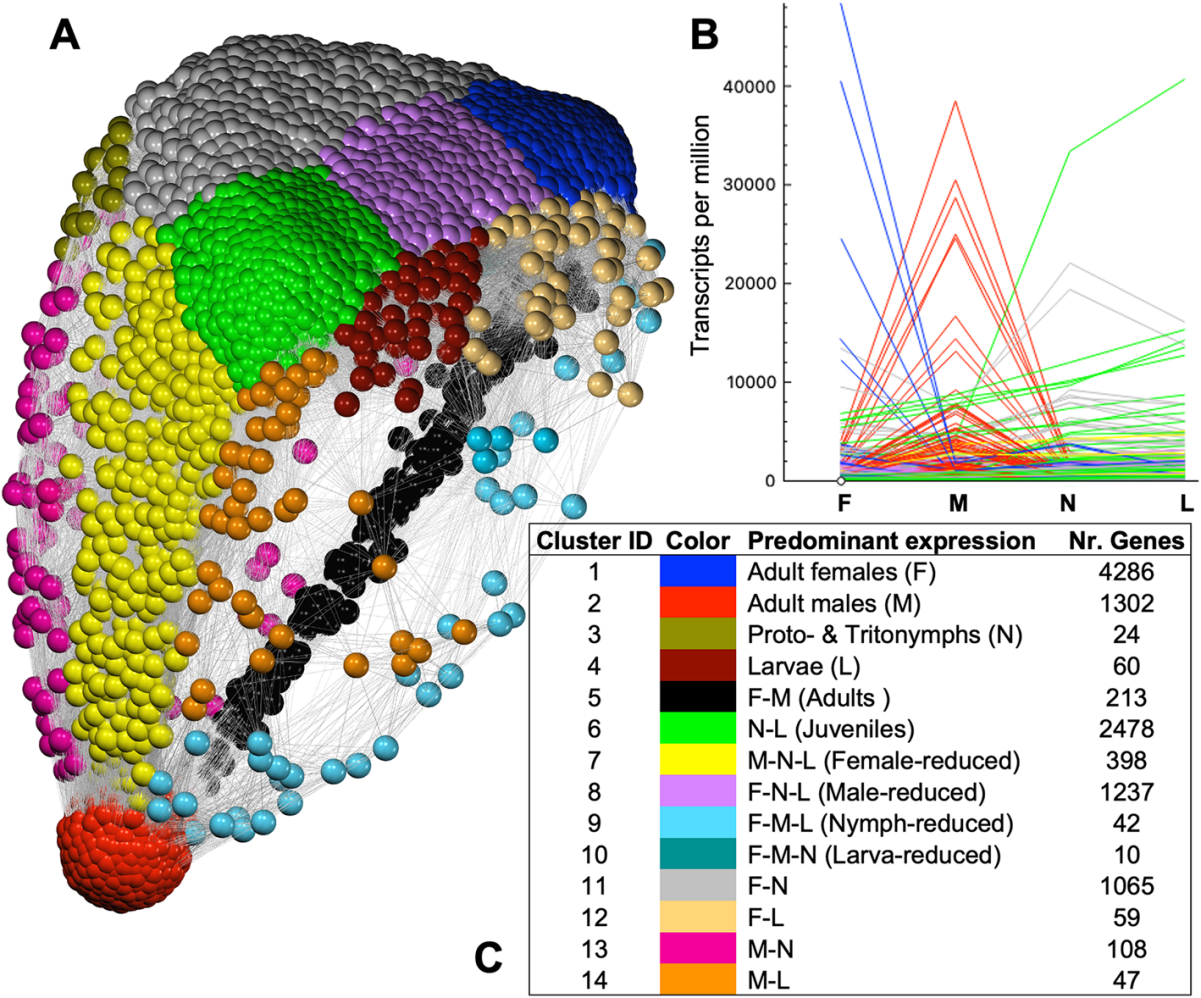
Network analysis of the *D. pteronyssinus* transcriptome. Panel A) Network graph of the complete transcriptome. Every point (node; *n* = 11,329, connected by > 10 M edges) corresponds to one gene and all nodes are distributed in three dimensions, with proximity depending on the similarity of the expression patterns of genes across stages (deduced from average transcript per million values using a Pearson correlation cutoff of 0.9). Individual genes were clustered using a Markov cluster algorithm with granularity value of 3.0, resulting in 18 clusters that were further curated based on similarity into 14 clusters (which are represented with different node colors). Panel B) Stage-specific gene expression profiles. The average TPM data (*n* = 3 replicated libraries) for all transcripts across the four analyzed stages is represented (F, M, N and L denoting females, males, nymphs and larvae, respectively); colors indicate individual gene clusters. Panel C) Description of the 14 gene clusters including names, color codes used in panels A and B, and gene numbers per cluster

Transcriptomic count data (average TPM from 3 replicates per stage) were used to generate a gene–gene network graph, resulting in 18 clusters that were further curated on the basis of similarity into 14 clusters, which included both single- and multi-stage predominant expression patterns (Fig. 2). The most abundant cluster corresponded to genes whose expression was predominant in females (*n* = 4286 genes), followed by genes whose expression was predominant in both larvae and nymphs (juveniles; *n* = 2478) and in males (*n* = 1302). The cluster assignments for each individual gene are listed in Additional file 2 (see headings to identify the appropriate column). This information was crosslinked with data on GO term annotation and *D. melanogaster* gene homologs*,* available for each gene (Additional file 2), to perform GO term and KEGG pathway functional enrichment analysis in a number of biologically relevant stage-dependent clusters with sufficient representation (i.e. predominantly expressed in adult females, adult males, both adults, both juveniles, and with reduced expression in females or males; see Fig. 2C). The significantly enriched GO terms (biological processes, BPs; molecular functions, MFs) and KEGG pathways for each cluster are summarized in Additional files 3 and 4, respectively.

The predominantly expressed genes in females (cluster 1) could be associated with numerous basal BPs (Additional file 3, tab “BP_Female_UP”), many of which are related to the active mitotic/meiotic status associated with oogenesis or early embryonic development, including *DNA* and *RNA metabolic processes* (GO:0006259 and GO:0016070, respectively, and related subprocesses), *regulation of cell cycle phase transition* (GO:1,901,987), and more general regulatory processes (GO:0009892, *negative regulation of metabolic process*). Other enriched BPs in this group were also related to an increase in cell division activity, as expected in reproductive tissues, including *cellular component organization or biogenesis* (GO:0071840) and *intracellular transport* (GO:0046907; comprising Golgi vesicle transport, nuclear transport or protein localization). A number of KEGG pathways [47] associated with these significantly enriched GO terms were also enriched among the genes predominantly expressed in females (Additional file 4, “KEGG_Female_UP” tab; see, for example, Additional file 1: Figs. S1, S2 and S3).

Male predominantly expressed genes (cluster 2) could be associated with a number of GO terms (Additional file 3, tab “BP_Male_UP”) related to male reproductive biology (as explained in more detail in the following section). Among the enriched GO terms we found categories related to proteolysis regulators (peptidases and their inhibitors; e.g. BP *proteolysis* GO:0006508, and MF *cysteine-type endopeptidase inhibitor activity* GO:0004869), hormone biosynthesis and signaling (e.g. BP *isoprenoid biosynthetic process* GO:0008299, and MF *hormone binding* GO:0042562), and several terms associated with other signaling regulatory mechanisms such as BP *protein phosphorylation* (GO:0006468; including kinase and phosphatase activities), *calcium-mediated signaling* (GO:0019722) or MF *semaphorin mediated binding* (GO:0030215). Additionally, two KEGG pathways were enriched among the predominantly expressed genes in males in our analysis: *fructose and mannose metabolism* (map00051) and *biosynthesis of amino acids* (map01230) (Additional file 4, “KEGG_Male_UP” tab).

The genes predominantly expressed in both juvenile stages (cluster 6) were related to different basal BPs, which could be compatible with an increased growth rate at these phases (Additional file 3, tab “BP_Juvenile_UP”), including the following: *generation of precursor metabolites and energy* (GO:0006091; along with several terms linked to the respiratory electron transport chain, ATP synthesis, etc.), *anatomical structure development* (GO:0048856; as well as terms involved in cell adhesion and multicellular organization), *monoatomic ion transport* (GO:0006811; and its regulation) and *regulation of cellular biosynthetic process* (GO:0031326; including several terms related to signaling, such as the BP *G protein-coupled receptor signaling pathway* GO:0007186, or the MF *DNA-binding transcription factor activity* GO:0003700). In agreement with a potentially greater energy generation activity in juveniles, the KEGG pathway *oxidative phosphorylation* (map00190) was also significantly enriched in this cluster (Additional file 4, “KEGG_Juvenile_UP” tab).

Finally, BP *actin cytoskeleton organization* (GO:0030036; and related terms) was significantly enriched in the cluster corresponding to genes with female-reduced expression (cluster 7), whereas the BP *peptide biosynthetic process* (GO:0043043; and related terms) was enriched in the male-reduced expression gene cluster 8 (Additional file 3, tab “BP_Female_DOWN” and “BP_Male_DOWN”, respectively). Further screening and discussion of individual genes and gene families related to the abovementioned processes/pathways are presented in the following sections. Genes lacking GO term or *D. melanogaster* homology data in our annotation as well as genes underlying biological processes not covered under the identified functional categories were also considered.

### Male reproductive processes

Previous comparative genomics and proteomic analyses of arthropod seminal fluid proteins (SFPs), as core components of the male ejaculate that are essential for reproductive success, revealed that the main functional classes generally associated with these proteins are conserved between taxa, although individual SFP genes can show signs of rapid and divergent evolution [62–64]. As described in the following subsections, potential *Dp* SFPs were identified by combining the sequence-based screening of putative orthologs of known arthropod SFPs and the assessment of expression profiles in annotated gene lists of functional groups commonly associated with SFP in the literature [65–70]. Finally, genes encoding other proteins that are overexpressed in males and potentially involved in the reproductive biology of *Dp* males are discussed in Sect. "Other proteins related to male reproductive biology".

#### Peptidases and peptidase inhibitors

**Fig. 3:**
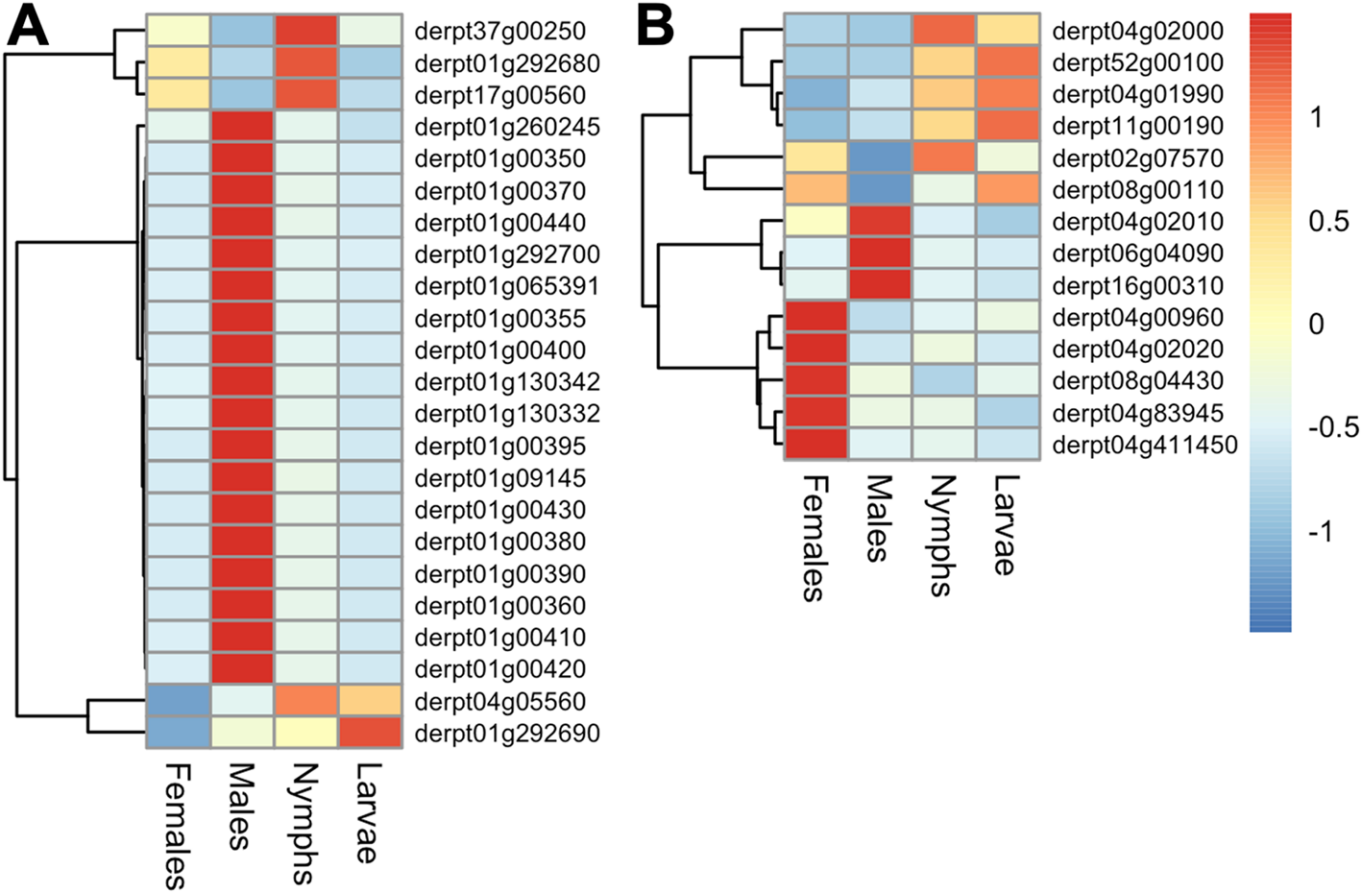
Stage-specific expression heatmap of selected peptidase inhibitor families in *D. pteronyssinus*. The darker red color indicates a higher transcript per million average (from *n* = 3 replicated libraries). Panel A) Expression level across stages of the 23 identified cystatin genes. Panel B) Expression of the 14 identified serpin genes. Gene IDs are indicated in each plot

The analysis of the 320 peptidase genes identified in *Dp* revealed significant overexpression in males over juveniles and adult females in genes of four families previously associated with SFP [66, 67, 69, 70]. These included 9 serine endopeptidases, 10 metalloendopeptidases, 8 cysteine endopeptidases and 1 peptidyl-dipeptidase (Additional file 2). All these genes presented moderate to low basal expression (TPM in males from 3 to 171.8), with the exception of the cysteine endopeptidase gene derpt21g00080 (TPM = 1739.2). Notably, 9 of these genes (including derpt21g00080) showed more than 50-fold overexpression in males compared to females. On the other hand, of the 65 peptidase inhibitor genes classified in the genome, male differential expression was detected in two families: 18 of the 23 identified cystatins (cysteine endopeptidase inhibitors) and 3 of the 14 serpins (serine endopeptidase inhibitors) (Additional file 2; Fig. 3). The TPM values of these genes in males ranged from 19.6 to 864.6 (for derpt01g09145), and 13 of the male-associated cystatin genes showed more than 50-fold overexpression compared to females (including derpt01g09145). Other serine peptidase inhibitor families previously found in seminal fluid, such as Kazal- and Kunitz-type inhibitors [67], were not overexpressed in *Dp* males in our analysis.

Peptidases and peptidase inhibitors are among the most represented and expressed classes of SFPs. The roles of serine endopeptidases (well represented among male-enriched peptidases in *Dp*), their serpin-like inhibitors, and metallopeptidases present in seminal fluids have been studied in detail in *D. melanogaster* [64]. The functions of these SFPs in Acari are less well known, but have also been identified among tick SFPs [66]. They are commonly involved in the activation or repression of proteolytic cascade pathways, leading to diverse physiological outcomes downstream, including roles in ovulation/egg production, sperm storage and fertility. The screening of potential orthologs of some of the well-characterized fruit fly seminal fluid peptidases of these families (e.g. the trypsin Sems, the trypsin inhibitor Acp62F, or the astacin metallopeptidase Semp1; CG10586, CG1262, and CG11864, respectively) yielded no results in *Dp*, suggesting that the male reproductive factors/strategies in mites may diverge from those of insects. In contrast to our knowledge of serine endopeptidases and serpin SFPs, the roles of cysteine endopeptidases and cystatins in arthropod reproduction are not well characterized, although some of them have been identified in the seminal fluid of *D. melanogaster*, ticks or mosquitoes [66, 67, 69]. In mammals, cystatins have been associated with sperm maturation [71]. Our transcriptomic data suggest that the identified cysteine endopeptidases and most of their cystatin inhibitors likely play prominent roles in the reproductive biology of *Dp* males, although further experimental evidence should shed light on their specific functions.

#### Immune proteins

Some SFPs can play protective roles against post-mating microbial infections of the female reproductive tract, either directly, as antimicrobial effectors, or by eliciting innate immune responses in females. An example of the latter is via the SFP hormone *sex peptide*, which regulates immune responses in *Drosophila* [62]. No ortholog was found in *Dp* for this gene; however, sex-biased differential expression patterns were detected in a number of genes potentially involved in *Dp* immunity. Notably, 4 out of the 6 antimicrobial peptide (AMP) genes of the previously annotated defensin family [25] were enriched in males. All of these genes showed more than 50-fold overexpression compared to females, including the two more basally expressed genes of the family (derpt66g00200 and derpt52g00130) (Additional file 2). The upregulation of defensins has also been detected in the accessory glands of ticks [66, 72], and has been associated with the protection of reproductive organs in insects and mammals [73, 74]. Additionally, 11 genes from a set of 24 putative AMP-like genes, previously annotated as cysteine-rich peptides [25], were also preferentially transcribed in males, with 11.7- to 71.3-fold greater expression in males than in females. Two genes encoding components of RNAi-related pathways also showed remarkable male-biased expression in our study: the argonaute protein Ago2 (derpt12g00670; 68.9-fold greater than in females) and an RNA-dependent RNA polymerase gene (derpt18g01450; 24.9-fold greater than in females). Ago proteins and Ago-bound small RNAs are essential for metazoan germline development and fertility, as has also been studied in insects [75].

#### Cell adhesion proteins

**Fig. 4:**
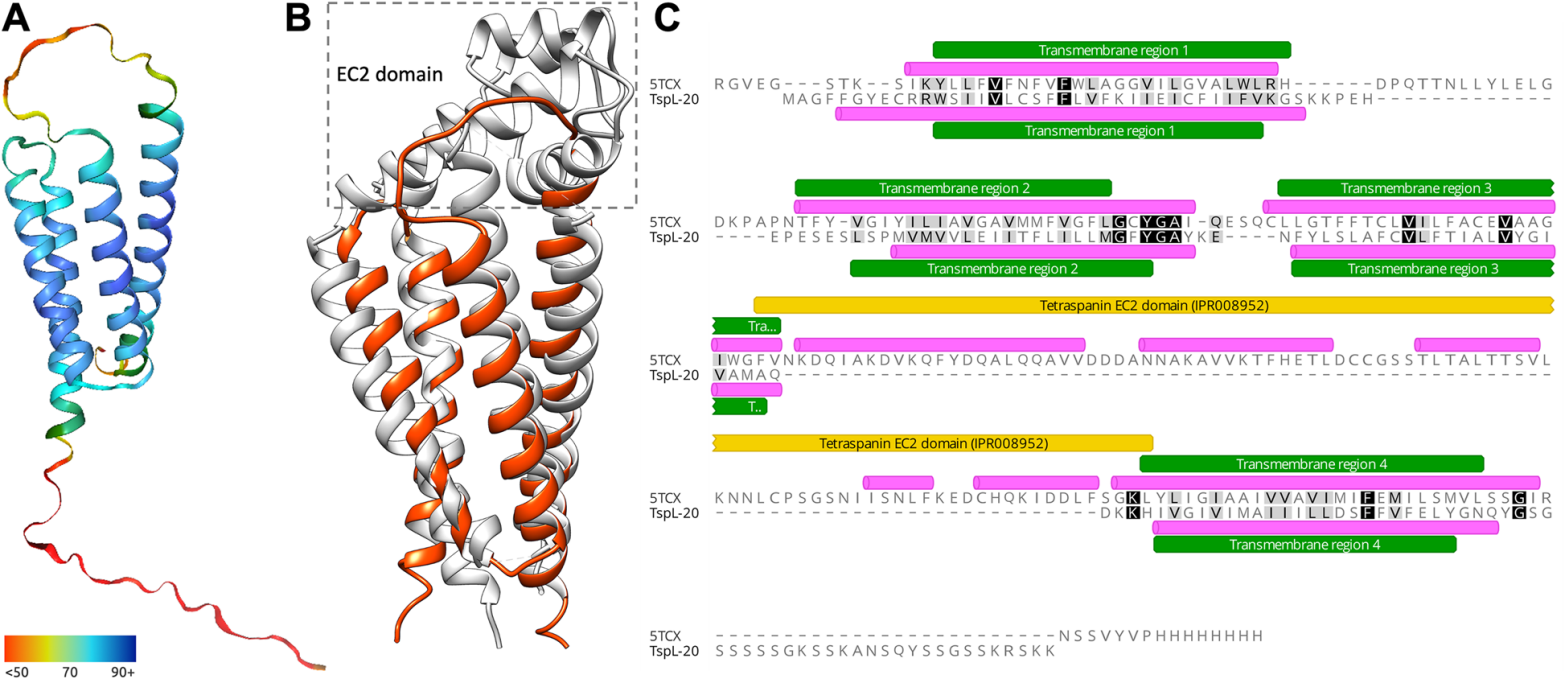
Structure alignment of the tetraspanin-like gene product derpt14g01940 with a human canonic tetraspanin**.** Panel A) Tetraspanin-like (TspL) derpt14g01940 structure prediction model using Alphafold 2 (Galaxy Version 2.3.1 + galaxy4). Colors indicate per-residue confidence scores (pLDDT) ranging from blue (highest) to red (lowest). Panel B) Superimposed structures of TspL derpt14g01940 (orange) and human tetraspanin CD81 (Protein Data Bank 5tcx) (grey). The C-terminal unstructured end of derpt14g01940 (24 aa) has not been depicted for visualization purposes. Panel C) Structure-based sequence alignment of TspL derpt14g01940 (TspL-20) and human tetraspanin CD81 (5tcx) as computed by PDBeFold, and visualized using Geneious Prime 2022.0.2 software (Biomatters Ltd., Auckland, New Zealand). Pink annotations denote alpha-helixes according model/structure; green annotations refer to transmembrane regions predicted by Phobius; the yellow annotation indicates the IPR008952 domain “Tetraspanin, EC2 domain superfamily”

Proteins involved in cell-to-cell adhesion, such as tetraspanins (Tsps), have been reported in transcriptomes/proteomes studying SFPs in different organisms, from mammals [76] to insects [69] and ticks [66]. One of the 19 Tsp genes annotated in *Dp* (bearing the tetraspanin domain IPR000301; Additional file 2) was strongly overexpressed in the male transcriptomes (Tsp-18, derpt07g00270; 59.8-fold greater than in females). The molecular structure of Tsps consists of four transmembrane alpha-helix domains, a small extracellular loop, and a large extracellular loop (domain EC2; IPR008952), which is important for the association with other proteins, creating membrane networks known as the *tetraspanin web* [77]. Additionally, a unique family of 23 tetraspanin-like protein (TspL) genes showing structural similarity to Tsps (Fig. 4A) was identified in *Dp*. These TspLs maintain the four transmembrane domains of Tsps but lack the conserved large extracellular domain EC2 (Fig. 4B and 4C). Interestingly, 16 of these TspL genes were notably overexpressed in males (with fold changes over females ranging from 9.2- to 204.8-fold). Orthologs of these TspLs could only be identified in other mites of the Sarcoptiformes order (data not shown). Canonic Tsps, and possibly TspLs, could participate in the reproductive biology of *Dp* males as constituents of extracellular vesicles abundant in seminal fluid, which have been associated with signaling processes leading to sperm maturation and fertility in other species [76, 78]. Other adhesion proteins related to seminal fluid/accessory glands in the literature, such as fasciclin or laminin [66, 67, 79], or those previously described in *Dp* [25] did not show noticeable associations with males in our transcriptomic survey.

#### Oxidoreductases

Up to 481 oxidoreductase genes from a number of enzyme families were annotated in *Dp* in this study (Additional file 2). Except for a catalase gene of low basal expression (derpt20g00080), none of the genes of the oxidoreductase families commonly associated with SFPs in the literature [65–69] were overexpressed in *Dp* males, including peroxidases (*n* = 23; comprising glutathione and thioredoxin peroxidases, as well as dual oxidases), glutathione S-transferases (*n* = 12) or superoxide dismutases (*n* = 9).

By regulating redox homeostasis and protecting against oxidative stress, antioxidant proteins are known to play key roles in the arthropod male reproductive physiology, from the maintenance of the testis germline [80] to the preservation of stored sperm fertility [65, 68, 81]. In line with their importance in reproduction, in *Dp* several genes from other oxidoreductase families were found to exhibit a clear male-enriched profile. Five out of the 7 pyrroline-5-carboxylate reductases (derpt08g03550, derpt19g01390, derpt06g03280, derpt11g00380, derpt21g00500) exhibited high expression (TPM = 155 to 608) and were markedly upregulated in *Dp* males (66.9- to 212.3-fold greater than in females). These genes encode the last enzyme in the L-proline biosynthesis pathway and are components of the functionally enriched KEGG pathway Biosynthesis of amino acids (map01230) cited in Sect. "Network analysis and functional annotation of stage-specific gene groups". Based on research in other animal systems, proline could play multifaceted roles in promoting sperm function/viability in *Dp*, particularly after long-term storage in the female *receptaculum seminis* organ. These roles include protection against oxidative stress and energy generation [82–85]. Similarly, 2 out of 5 sorbitol dehydrogenase (SORD) genes (derpt22g01060 and derpt18g00050) exhibited male-enriched transcription (7.7- and 156.7-fold greater than in females, respectively). SORDs are implicated in the catabolism of sorbitol and other sugar alcohols and are included in the functionally enriched KEGG pathway map00051 (Sect.  "Network analysis and functional annotation of stage-specific gene groups"). In *D. melanogaster*, the SORD gene CG4836 shows testis-specific expression [86, 87], yet its specific role has not been described. As discussed for proline, SORD could also play a role in energy metabolism by converting sorbitol to fructose and generating the NADH needed for ATP generation. Interestingly, SORD has been found in the flagella of mouse spermatozoa, where sorbitol is used as fuel for sperm motility [88]. A similar function could be expected for *Dp*; although its sperm is aflagellate [1] and the sperm motion mechanism still needs to be elucidated.

Other male-regulated oxidoreductase genes were included in the cytochrome b561/cytochrome b reductase 1 family (containing the IPR043205 domain), where 3 out of the 6 annotated genes were overexpressed in males (derpt38g00200, derpt11g01630, derpt38g00160), ranging from 31.1- to 104.9-fold overexpression compared with females. These transmembrane enzymes contribute to the ferric ion/ascorbate homeostasis, although little is known about their function in insects [89]. Some of these genes display restricted expression patterns in *Drosophila*, such as CG3592, which is mostly expressed in the male testis [86, 87], denoting possible unexplored links with reproduction. Finally, of the 40 cytochrome P450 (CYP) genes identified in the genome, 4 were significantly overexpressed in males (derpt12g00440, derpt06g02690, derpt10g0600545, derpt18g00350; ranging from 3.4- to 47.6-fold greater than in females). The encoded proteins do not show homology to conserved CYPs that participate in the ecdysteroid or juvenile hormone biosynthetic pathways (see Sect. "Hormone and pheromone metabolism, signaling and transport"), although a potential role in the metabolism of signaling messengers cannot be excluded. For instance, in the biosynthesis of poorly studied monoterpenoid aggregation/sexual pheromones [1, 90]. Aside from this possibility, studies detecting the expression of insect CYPs in seminal fluid or in accessory glands have suggested that they may exert an antioxidant/detoxification protective function that promotes fertility [68, 91].

#### Lipases

The most commonly reported families of SFP lipases include triacylglycerol lipases, which are essential for sperm quality/quantity and are transferred into females during copulation to provide energy for sperm motility by hydrolyzing triglycerides [92, 93], and phospholipases, which could be related with the biosynthesis of prostaglandin E2 [66]. A screen of previously annotated *Dp* lipases (Additional file 2) [29] revealed significant overexpression in males of 3 triacylglycerol lipase genes (EC 3.1.1.3; with derpt07g315475 being overexpressed 49.3-fold compared with females) and 9 phospholipase A2 genes (EC 3.1.1.4; up to 98.3-fold versus females for derpt38g00100).

Besides these genes, we found an expansion of 10 enolase-phosphatase E1-like genes in the *Dp* genome (acireductone synthases, EC 3.1.3.77; domain PTHR20371), 9 of which were overexpressed in males (from 11.9- to 103.3-fold in males versus females). A similar result was found when we analyzed previously published transcriptomic data on *Psoroptes ovis* [34]. Moreover, inspection of the OrthoMCL orthology group OG6_104759 [94], which is associated with this family, revealed that insects generally possess a single or only a few genes bearing this domain (e.g. *Drosophila* CG12173; *n* = 1), whereas it appears to be expanded in other Acari species, such as *Sarcoptes scabei* (*n* = 10) and *Leptotrombidium delicense* (*n* = 11). Enolase-phosphatase E1 is known to form part of the methionine salvage pathway, which is related to fecundity in *Drosophila* [95], but the reasons for their expansion in mites and their specific role in male physiology need to be further explored. Additionally, genes encoding 1-alkyl-2-acetylglycerophosphocholine esterases (EC 3.1.1.47) were also significantly enriched in males (3 out of the 6 annotated genes; 42.1- to 64.4-fold greater than in females). These enzymes participate in the metabolism of ether lipids that are particularly abundant in human sperm [96]. Other functionally undescribed lipase genes exhibiting male-related expression included acid phosphatase derpt02g06050 and alkaline phosphatase derpt01g292030. Finally, we identified 24 male-related moderately expressed protein-serine/threonine phosphatase (EC 3.1.3.16) genes potentially linked to signal transduction and regulation.

#### Glycosylases

In general, our transcriptomic analysis of glycosylase genes revealed discrete expression differences between stages/adult sexes. Only two genes, derpt15g0455375 and derpt18g00250, presented slightly increased expression in males compared with juveniles and females. On the other hand, the screening of known SFP glycosylases from other species identified one beta-N-acetylhexosaminidase (EC 3.2.1.52; derpt22g00330) with homology to *Drosophila* Hexosaminidase 2 (CG1787), which exhibited significantly greater expression in males than in females (14.1-fold) and nymphs (2.3-fold), but not in larvae. This protein has been reported as a SFP both in insects [67] and in humans [97].

#### Other proteins related to male reproductive biology

Three ubiquitination-related enzymes were significantly enriched in the male transcriptome: two conjugating enzymes (derpt06g04000, derpt20g00540), the first being highly basally expressed (TPM = 1258) and the second showing an acute male-enriched expression pattern (107.7-fold greater than in females), and one ligase (derpt18g293450). Ubiquitination mediates protein degradation via the ubiquitin–proteasome pathway, which is essential for maintaining the homeostasis of regulatory proteins that participate in diverse biological processes, including reproduction [98, 99]. Moreover, ubiquitination-conjugating enzymes have been found among female-transferred SFP in *D. melanogaster* [100] and are highly expressed in male reproductive tissues and spermatophores of ticks [66].

Protein phosphorylation mediated by kinases also plays a critical role in the regulation of spermatogenesis. A total of 258 protein kinases (PKs) were annotated in the *Dp* genome in this study on the basis of KEGG annotations and homology to *D. melanogaster* (Additional file 2), 52 of which were overexpressed in males, suggesting possible reproductive roles. The majority of these genes were homologous to three PK families. First, all 17 annotated testis-specific serine kinase-like (TSSK) genes were enriched in males (from 3.6- to 163.6-fold overexpression compared with females). In humans, six TSSKs are known to be expressed in the testis and function in spermiogenesis, the last phase of spermatogenesis involving sperm maturation [101]. In *Drosophila,* two TSSK-like genes have been identified (CG14305 and CG9222), both of which are also predominantly expressed in the testis [86, 87], whereas in the tick *Dermacentor variabilis,* at least two TSSKs have been detected in male reproductive tissues but not in spermatophores, which indicates that these proteins are not transferred to the female during copulation [66]. The analysis of the OrthoMCL group OG6_103794, associated with TSSKs, indicated that other mites, including *S. scabei* (*n* = 13) and *L. delicense* (*n* = 10), also presented similar expansions of these genes. The second PK family, casein Kinase 1-like (CK1), includes 10 annotated genes in *Dp*, 7 of which were enriched in males (27.1- to 67.6-fold greater than in females), and 5 of which encode homologs of *Drosophila* gilgamesh (CG6963), a PK required for germline stem cell maintenance in the testis [102]. The third family includes 5 haspin-like PK genes, all of which exhibited strong male transcription (21.4- to 170.4-fold greater than in females). In *Drosophila,* haspin CG40080 is involved in histone phosphorylation during mitosis, but, unlike *Dp*, its expression has not been associated with male tissues [86, 87].

In view of the importance of regulatory mechanisms in reproduction, as well as the possibility of finding targets for the design of acaricides among these processes [12, 71, 103, 104], additional broadly recognized families of genes associated with regulatory and signaling pathways were further annotated in *Dp* to detect possible male-associated factors. These included transcription factors (TFs), protein signaling messengers (cytokines and neuropeptides), cytokine receptors and G protein-coupled receptors (GPCRs), as well as small GTPases and their associated proteins. The biosynthesis of hormones, a non-protein class of signaling messengers, has been addressed separately in Sect. "Hormone and pheromone metabolism, signaling and transport".

A total of 367 genes encoding potential TFs were identified (Additional file 2), 11 of which were significantly overexpressed in males over other stages, particularly homeodomain TF derpt34g00200, Fork Head Box TF derpt12g01310, and high mobility group box domain TF derpt08g04670. Our results suggest a link between these genes and male reproduction or development, although in most cases, clear homology to well-described specific TFs from other species could not be found. In the case of derpt08g04670, homology was detected to Sox100B (CG15552), which is essential for male-specific gonad development in *Drosophila* [105]. Among the TFs, we identified 31 genes coding for nuclear receptors (NRs) of different families (Additional file 2), a specific class of TFs that bind hormones or other lipophilic molecules known to regulate development and reproduction [106]. The number of NRs identified herein is in agreement with previous reports on other mites (*n* = 30 in *Tetranychus urticae*) [33], although none of them were differentially expressed in *Dp* males. Genes encoding signaling proteins were also classified, and 23 neuropeptides and 6 cytokines were identified (Additional file 2). Only three of these genes, a cytokine (derpt71g00060) and two neuropeptides (derpt16g02480, derpt02g00140), which have unknown functions in male biology, were significantly overexpressed in males. Among their possible receptors, 1 cytokine and 92 GPCRs (Additional file 2), only 2 showed significantly higher (but basally low) expression in males (derpt25g00640, derpt13g02280). For small GTPases, none of the 62 annotated genes were significantly overexpressed in males, while 6 out of the 200 genes encoding GTPase-associated proteins were upregulated (Additional file 2). Among them, we detected two Rho GTPase-activating protein genes showing homology to *Drosophila tum* (CG13345), which is involved in Wnt signaling regulation (derpt05g00900 and derpt01g01500; the latter being highly male specific: 47.4-fold greater than in females), as well as a calmodulin-encoding gene (derpt12g01350) related to the calcium signal transduction pathway.

Among the 3 arginine kinase (AK) genes annotated in the genome (derpt02g08050, derpt22g00730, and derpt31g00440), the expression of the latter was strongly associated with males (120.3-fold greater than in females). Considering the essential function of AKs in invertebrates, which contribute to sustaining cellular energy metabolism via the biosynthesis of phosphagen and are especially abundant in muscle tissues [107], a possible specialization of this gene in males could be suggested. For example, it could support the production and delivery of sperm, which are highly energy-demanding processes. In agreement with this hypothesis, 2 of the 4 described AKs in *Drosophila* (CG5144 and CG4546) are almost exclusively expressed in male testes [86, 87]. In addition, high AK activity in the abdomens of reproductive males has been associated with mating activity in ants [108].

Finally, among the male-overexpressed genes, a remarkable number of *Dp* genes of unknow function presented poor homology in databases. This was particularly pronounced in the case of genes encoding small proteins (e.g. up to 100 amino acids). Approximately 30% of these genes in the genome (157/521) presented significantly greater expression in males than in females, whilst only 6% (33/521) presented the opposite profile. Some of these genes were over 100-fold overexpressed in males compared with females (derpt41g346090, derpt19g00020, derpt03g05480, derpt03g06140, derpt01g10000, derpt34g00630 and derpt02g03690). Other uncharacterized genes encode proteins whose sequence attributes are compatible with those of cuticular proteins (as detailed in Sect. "Cuticular proteins"). Additionally, a previously undescribed family was identified, with at least 119 genes showing 2.7- to 429.7-fold greater expression in males compared to females. This family encodes proteins with a conserved structure (as predicted by Alpha-fold) generally consisting of an N-terminal transmembrane alpha-helix, followed by a four-blade beta-propeller non-cytoplasmic domain (predicted to be outside the membrane), one or more transmembrane alpha-helices, and a C-terminal putatively disordered cytoplasmic domain of variable length (Additional file 1: Fig. S4). No other domains have been detected in these proteins, whose genes have been designated as “male specific four-bladed beta-propeller fold family” (4BetaP) (Additional file 2). Four-bladed propeller structures in the literature are generally associated with substrate binding and transport [109]. The *Caenorhabditis elegans* proteins Q22625 and Q19393 (UniProt IDs), both of which are expressed in the germline, share structural similarity with derpt27g00030 (Additional file 1: Fig. S4A), but their physiological role is unknown.

### Female reproductive processes

DEG and network analyses have shown that female-associated genes are the most prevalent expression profile in *Dp* (Fig. 1B and 2C, respectively). Herein, we explore the female-enriched transcriptome to identify genes with a reported link with oogenesis and vitellogenesis, but also genes of functional groups showing potential association with female reproduction, including peptidase regulators, oxidoreductases and other signaling factors.

#### Genes with a known role in oogenesis

As determined by GO term and KEGG pathway functional enrichment analyses, a number of categories enriched in the female-upregulated transcriptome were related to DNA replication and repair (Sect. "Network analysis and functional annotation of stage-specific gene groups"). These findings are consistent with the importance of such processes for meiosis during gametogenesis, as well as for mitotic division in early embryos (still within the sampled gravid females) or in stem cells of the ovarian germline. Potential orthologs of well-studied genes showing upregulation in ovaries in *Drosophila* and participating in mitosis/meiosis [110], such as Aurora kinases (derpt12g01360 and derpt27g00350), or contributing to early oocyte/embryo development, like nanos-type kinases (derpt19g00270), were significantly overexpressed in *Dp* females in our study. Other GO term and KEGG pathway categories associated with females were related to RNA processing. The upregulation of genes associated with RNA biology could be explained by the need to regulate mRNA localization, degradation and translation in oocytes and early embryonic development. In this context, among the most highly expressed genes in the female transcriptome, we detected the RNA helicase gene derpt06g00100 (the top 26 expressed genes by TPM), which is a homolog of the *Me31B* (CG4916) gene in *Drosophila*, and derpt22g01320 (top 46) similar to *tral* (CG10686). Both genes are essential for mRNA localization in *Drosophila* oocytes [111].

Our survey of oogenesis-related genes in *Dp* was further extended under the premise that the molecular machinery for oogenesis is substantially conserved across species [112]. For that purpose, *D. melanogaster* homology data for each *Dp* gene (Additional file 2) were mapped against known fruit fly accessions showing ovary-associated expression according to the FlyAtlas 1 survey dataset [86, 110]. In addition, KO term annotations for each *Dp* gene were cross-linked with KO terms included in oogenesis-related KEGG pathways (Oocyte meiosis, map04114; Cell cycle, map04110). This screening strategy yielded a total of 351 putative oogenesis-related genes, 131 of which were significantly overexpressed in *Dp* females in all pairwise comparisons (Additional file 2). A common gene family among female-associated genes was protein kinases (*n* = 9) which, together with phosphatases such as M-phase inducer phosphatase (derpt04g02240), are known to tightly regulate the different phases during mitosis and meiosis [113, 114]. In addition to the two Aurora kinases cited above, other female-upregulated PKs performing similar functions included polo-like kinase (derpt13g00110), cell division control protein 7 (derpt01g08670), mitotic spindle checkpoint protein Bub1/Mad3 kinase (derpt33g00490) or cyclin-dependent kinase 2 (derpt07g01010). Other functional groups putatively associated with these regulatory processes and upregulated in females included cyclins (derpt01g07480, derpt36g00330, derpt24g00370, derpt03g04570), other cell division control proteins (derpt01g03060, derpt19g00460) and DNA replication licensing factors (derpt03g03480, derpt14g00820, derpt32g00540, derpt15g01670, derpt07g02390, derpt01g03550). In addition, a number of genes encoding putative cytoskeletal components conforming the mitotic and meiotic microtubule spindle [115, 116] were significantly upregulated in females; including genes encoding tubulins (derpt05g00300, derpt04g06020), kinesins (derpt02g03950, derpt36g00340, derpt13g01190, derpt18g01610, derpt24g00430), dynactins (derpt26g00310, derpt12g00880, derpt32g00360), the microtubule-associated protein MAP65/Ase1/PRC1 (derpt02g00970), and Ndc80 and Nuf2-like kinetochore proteins (derpt04g05130 and derpt06g00240, respectively).

#### Vitellogenesis and lipid metabolism

Vitellogenesis is a common process in oviparous animals throughout very distinct phyla and consists in the uptake of vitellogenin, the lipid-binding glycoprotein that acts as a precursor of egg yolk, into developing oocytes by receptor-mediated endocytosis [117]. Some of the critical genes involved in this process were identified in *Dp*: 3 vitellogenin-like apolipoprotein genes (derpt07g03520, derpt07g02270, derpt01g01310) and a putative vitellogenin receptor (derpt16g00410) homologous to *Drosophila yolkless* (CG1372). All of these genes exhibited strong differential expression profiles in female mites (from 7.3- to 26.9-fold greater than in males), with the first two being among the top 50 expressed genes as per TPM. In addition, a number of genes involved in lipid metabolism were upregulated in females, indicating the need to supply nutrients to highly demanding reproductive tissues and to build up lipid reserves in oocytes. These genes include a highly expressed perilipin-like gene (derpt24g01290; top 49 by TPM), which could participate in the formation of lipid droplets on the basis of the function of its *Drosophila* homolog *Lsd-2* (CG9057); an acetyl-CoA synthetase-like gene (derpt01g04190; top 80) putatively regulating fatty acid biosynthesis; and four lipase-encoding genes predicted to hydrolyze triglycerides (derpt86g00040, derpt13g01470, derpt04g06190, derpt86g00050).

#### Peptidases, peptidase inhibitors and oxidoreductases

Gene families previously highlighted in the context of male-specific processes and not included in the list of oogenesis-related genes described above also included genes with a distinct female expression pattern, indicating possible roles in reproduction. Twenty-three peptidases, including mostly serine, cysteine and metallo-endopeptidases (*n* = 5, 5 and 4, respectively), were significantly overexpressed in females compared with other stages (Additional file 2). No clear association with known oogenesis-related peptidases was found for the three most highly overexpressed genes in females, two cysteine (derpt18g01460, derpt18g01470) and one serine (derpt77g00050) endopeptidase genes (14.1-, 13.6- and 11.8-fold greater than in males, respectively). However, possible functions could be inferred for other genes on the basis of homology. The derpt26g00300 gene encodes a separase-like cysteine endopeptidase that plays a key role in chromosome segregation during anaphase [118]. Three homologs of well-described metalloendopeptidase genes of *Drosophila* were also found: derpt04g179450, derpt14g00260 and derpt04g05310. The first of these genes shows homology to the *maternal haploid* gene (CG9203)*,* which is essential for the integrity of paternal chromosomes during the first zygotic mitosis [118]. The second is an ADAMTS-like peptidase gene similar to *stall* (CG3622), which participates in the formation of the ovarian follicle [119]. The third gene encodes a homolog of neprilysin 2 (CG9761)*,* a peptidase involved in early embryonic development and required in the fly spermathecae for fecundity [120]. Among the less characterized peptidases, metallocarboxypeptidase derpt07g01560 merits further study since it was the third most highly expressed peptidase gene in females in our analysis (TPM = 1824) and showed strong differential expression in females (8.8-fold greater than in males).

We identified 4 serpins that were differentially expressed in females (Fig. 3B). Among them, derpt04g02020, encoding the serpin allergen Der p 27 [3], was remarkably expressed in females (the top 18 most highly expressed gene by TPM in the female transcriptome). Interestingly, a recent transcriptomic study revealed a similar result in females of the parasitic mite *P. ovis* regarding its Der p 27 ortholog [34]. These results suggest a potential role for the identified mite serpins in reproduction and/or embryogenesis. Serpins have been implicated in the regulation of many biological processes in arthropods, from innate immunity and host–pathogen interactions to development and reproduction [121]. In *Drosophila*, serpin27A has been shown to regulate embryonic axis formation via the inhibition of a serine peptidase cascade linked to the Toll pathway [122].

Several oxidoreductase genes were significantly overexpressed in females (*n* = 34; Additional file 2). Genes of this family have recently been reported to be overexpressed in females of the poultry red mite *Dermanyssus gallinae* and associated with mite reproductive and/or nutritional activities [35]. Among *Dp* oxidoreductases with a reported link with reproduction/development, derpt04g05410 encodes a ribonucleoside diphosphate reductase large subunit involved in DNA biosynthesis, whose upregulation in females is consistent with the high mitotic activity expected during oogenesis, as discussed above. In addition, a glucose dehydrogenase (derpt24g01100) linked to female fertility in insects [123] was also overexpressed, as were two cytochrome P450s that participate in the ecdysteroid hormone biosynthetic pathway (derpt04g04900 and derpt10g00710), as described in Sect. "Ecdysteroid hormones biosynthetic pathway".

#### Other signaling factors

As indicated for PKs, phosphatases or some proteolytic regulators and following the approach presented for male reproductive processes, female-associated genes from additional families commonly involved in regulation and cell signaling were also evaluated. Most of these genes remain poorly described in mites, and their role in female biology needs to be further elucidated; the different categories are outlined below. Three ubiquitination-related enzymes were significantly enriched in females: two conjugating enzyme-encoding genes (derpt33g00510, derpt38g00070), the first of which was 9.2-fold more highly expressed in females compared with males, and one ligase (derpt14g00650). Several TF genes were significantly differentially expressed in females (*n* = 32; Additional file 2). Some of these genes have already been identified via the above-described screening strategy for oogenesis-related genes. These included two genes encoding ATRX-like transcriptional regulators: derpt43g00220, a homolog of *Drosophila* XNP (CG4548) potentially involved in chromatin organization [124]; and derpt66g00110, a homolog of okra (CG3736), which functions in DNA repair during meiotic recombination [125]. In addition, we identified two TATA box binding protein (TBP) transcription initiation factor genes (derpt16g02150, derpt33g00600), which are components of the female-enriched KEGG pathway “basal transcription factors” (map03022; Additional file 4, “KEGG_Female_UP” tab), whose homolog in *Drosophila*, *Tbp* (CG9874), is upregulated in ovaries [110]. Other nonpreviously listed TF genes included derpt02g03630, which encodes a capicua-like transcriptional repressor that participates in early embryogenesis in *Drosophila* [126]; an unclassified NR of unknown hormone receptor ligand and function (derpt15g00150); and a nuclear protein 1-like gene (derpt05g03420) of unknown function showing very high expression in females (TPM = 1101; 9.5-fold overexpression compared with males). One cytokine-like gene (derpt71g00050) and two neuropeptide genes (derpt11g01690 and derpt03g364630) were also overexpressed in females (Additional file 2). The latter genes encode, respectively, crustacean hyperglycemic hormone-like (CHH) and ecdysis triggering hormone-like (ETH) neuropeptides, which, although being canonically related to the molting process (see Sect. " Regulation of hormone biosynthesis and transport") [106], could also play a role in female reproduction, as observed for different ecdysis-related neuropeptides in insects [127]. In contrast to these two genes, approximately half of the identified neuropeptide genes (*n* = 12) were significantly underexpressed in females compared with other stages, which suggests different but undescribed regulatory functions in females. Similarly, of the 27 GPCR-like receptor genes that were differentially expressed in adult females, 25 were repressed in females compared with other stages (Additional file 2). These include 13 receptors of the rhodopsin family (potentially binding neuropeptides), such as the product of derpt07g03610, which, together with derpt07g03360 (which is overexpressed in females), shows homology to the SIFamide receptor (CG10823) required to determine sex mating behavior in *Drosophila* [128]. Among the 4 small GTPase genes overexpressed in females (Additional file 2), 3 show poor homology to characterized GTPases (derpt05g00260, derpt36g00420, derpt17g00120), whereas one (derpt37g00540) shows homology to *Drosophila* Rab30 GTPase (CG9100), which is involved in JNK signaling during embryogenesis [129]. In addition to small GTPases, 22 GTPase-associated protein genes were enriched in females, including 3 genes encoding Rho GTPase-activating proteins (derpt09g00960, derpt27g00450, derpt05g02640) homologous to the ovary-expressed *tum* gene from *Drosophila* (CG13345) [86, 110]. Among the four GPCR gustatory receptor-like proteins annotated in the genome (according to KEGG nr. K08471; Additional file 2), our analysis identified one gene (derpt34g00220) with reduced expression in females, which could be involved in sex-dependent communication/perception. Also potentially linked to chemosensory processes, we identified 5 tandemly arranged genes (derpt03g00430, derpt03g00440, derpt03g00450, derpt03g364600 and derpt03g365150) which are still to be characterized. These genes exhibit notable female overexpression (ranging from 6.2- to 33.3-fold versus males), which, despite lacking sequence homology to any other species in databases, show moderate structural homology to insect odorant/pheromone-binding proteins (e.g. PDB accessions 4pt1 and 3cab; as computed for derpt03g00440 via the Phyre2 server; see LOC113794088 at the AlphaFold Protein Structure Database, corresponding to derpt03g00450, for a predicted structure of these proteins).

### Hormone and pheromone metabolism, signaling and transport

Our knowledge of the underlying molecular mechanisms associated with two main arthropod endocrine hormone groups, ecdysteroid hormones (e.g. 20-hydroxyecdysone, 20E, in insects) and sesquiterpenoid hormones (e.g. juvenile hormone III, JH III, in insects), remains limited in Chelicerata [130–132] and has not been addressed in *Dp*. In this section, we inspect the genes involved in the biosynthesis, signaling activity, regulation and transport of both hormone groups and determine their transcription levels across stages. Additionally, genes potentially involved in the biosynthesis of terpenoid pheromones are discussed.

#### Ecdysteroid hormones biosynthetic pathway

**Fig. 5:**
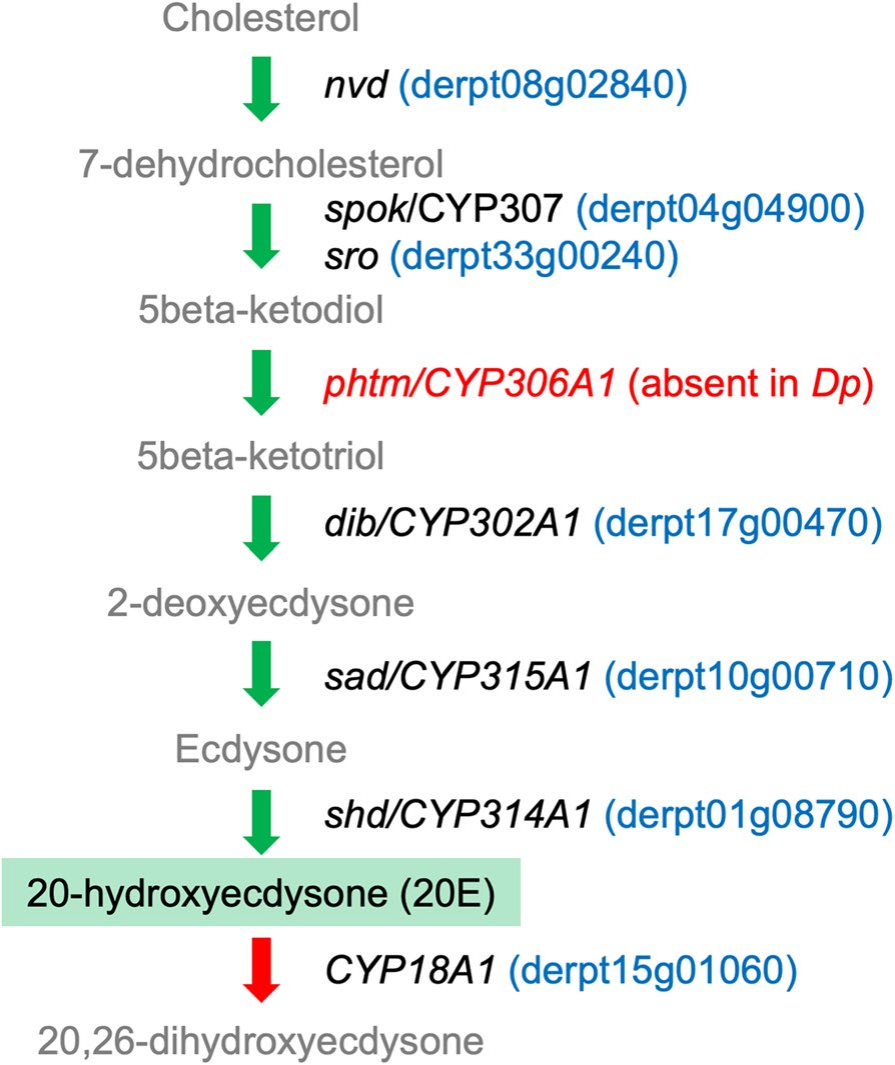
Ecdysteroid biosynthetic pathway. Canonic ecdysteroid hormone intermediates and bioactive 20E (highlighted in green) are depicted together with the names of *Drosophila melanogaster* genes encoding enzymes for their conversion: *dib* (disembodied), *nvd* (neverland), *phtm* (phantom), *sad* (shadow), *shd* (shade), *spok* (spookier), *sro* (shroud). Green arrows indicate biosynthesis and red arrow indicates inactivation. *Dermatophagoides pteronyssinus* (*Dp*) gene orthologs are shown in blue in brackets

As learned from insect model systems, the titers of each hormone group (i.e. their homeostasis) can be coordinated by controlling their respective metabolic pathways. We identified gene orthologs for all enzymes of the ecdysteroid biosynthesis pathway, the so-called “Halloween genes”, except for *Phantom* (*phtm*; *Drosophila* CG6578), which encodes the C25 hydroxylase enzyme CYP306A1 (Fig. 5; Additional file 2). This gene has not been detected in Chelicerata, suggesting that differentially modified ecdysone molecules other than 20E are to be bioactive in this phylum [106]. This has been confirmed in *T. urticae,* where ponasterone A (25-deoxy-20-hydroxyecdysone) has been detected in the absence of 20E [33]. A similar scenario could be expected for *Dp* on the basis of the molecular components identified in the pathway of both species. Unlike *T. urticae, Dp* also expresses a gene ortholog of the CYP18A1-like protein, a C26 hydroxylase/oxidase involved in hormone inactivation [133]. Inspection of our transcriptomic data revealed that i) the orthologs of *spok* (derpt04g04900) and *sad* (derpt10g00710) were the only P450 genes in the genome (*n* = 40), together with vitamin D 25-hydroxylase-like derpt33g00060, which were overexpressed in females in all pairwise comparisons; ii) the ortholog of *shd* (derpt01g08790), a gene coding for a CYP314A1-like enzyme putatively finalizing the synthesis of bioactive hormones (e.g. ponasterone A), was enriched in juveniles (larvae and nymphs); and iii) the CYP18A1-like gene, potentially involved in ecdysteroid inactivation, showed significantly lower expression in females. The differential expression of genes of this pathway across stages/sexes could be related to the different physiological roles attributed to ecdysteroids during development, which can contribute to regulate from molting [106] to reproductive maturation and vitellogenesis [134]. Indeed, transitions between molting phases in mites are known to be controlled by sequential peaks of bioactive hormones [132]. Compared with the cited study, the determination of the expression dynamics of these genes across stages in our analysis might be limited by the sampling method, which did not include non-motile juveniles (molting pharate stages), mixed proto- and tritonymphs, and did not synchronize the developmental status of the pooled mites.

#### Sesquiterpenoid hormones biosynthetic pathway

**Fig. 6:**
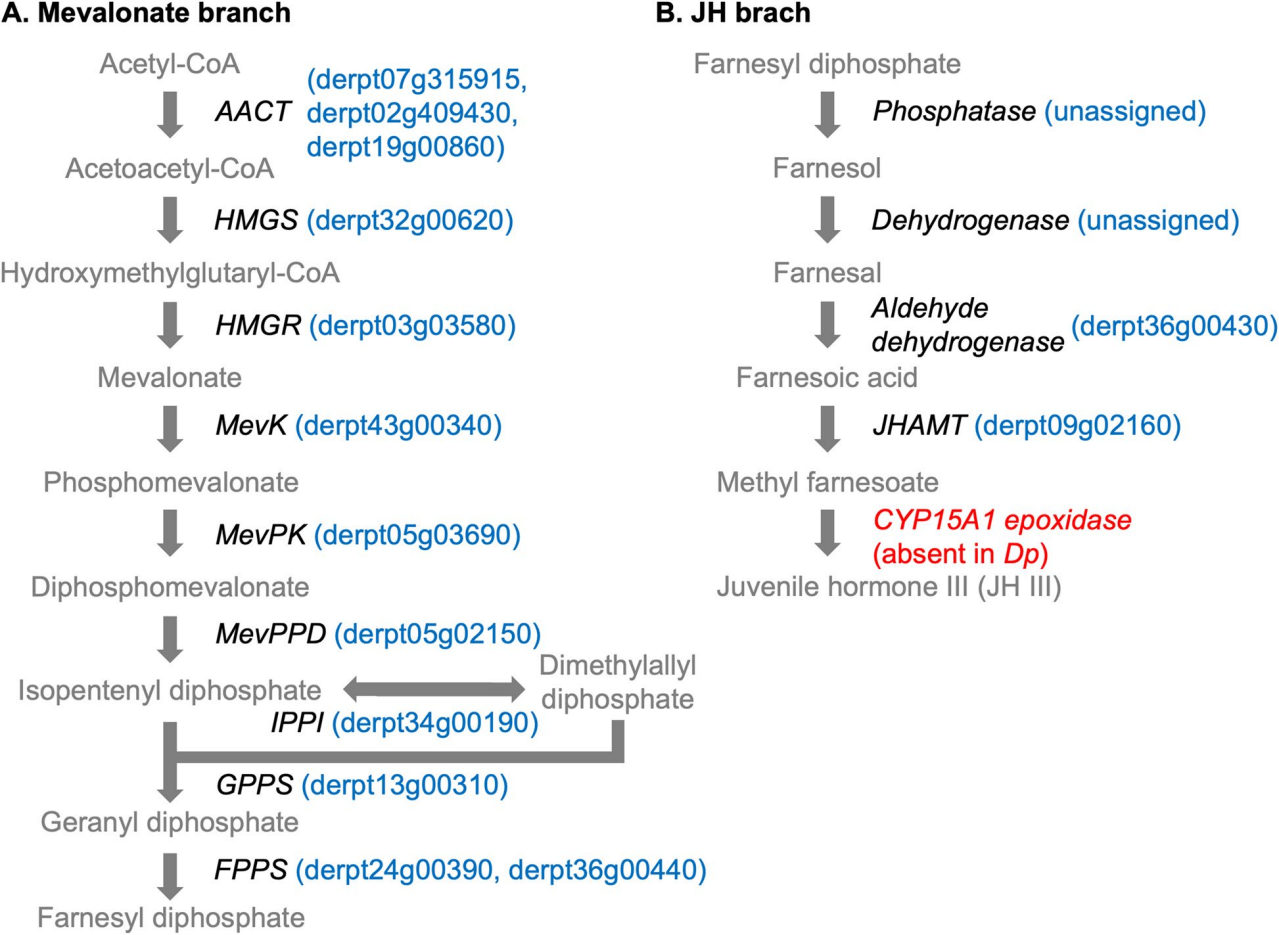
Sesquiterpenoid biosynthetic pathway. Intermediate products in the early canonic mevalonate pathway (Panel A) and the downstream juvenile hormone (JH) branch of insects (Panel B) are depicted together with the names of the genes encoding enzymes for their conversion: *AACT* (acetoacetyl-CoA thiolase), *HMGS* (hydroxymethylglutaryl-CoA synthase), *HMGR* (hydroxymethylglutaryl-CoA reductase), *MevK* (mevalonate kinase), *MevPK* (phosphomevalonate kinase), *MevPPD* (diphosphomevalonate decarboxylase), *IPPI* (isopentenyl diphosphate isomerase), *GPPS* (geranyl diphosphate synthase), *FPPS* (farnesyl diphosphate synthase), *JHAMT* (juvenile hormone acid methyltransferase). *Dermatophagoides pteronyssinus* (*Dp*) gene orthologs are shown in blue in brackets

Inspection of the sesquiterpenoid juvenile hormone (JH) biosynthesis pathway led to the annotation of putative orthologous genes for at least 11 of the 14 enzymes that lead to JH III synthesis according to the canonic pathway, which is divided into two parts: the mevalonate branch and the JH branch [130] (Fig. 6; Additional file 2). By mapping to the KEGG pathway map00900, we found orthologs for all genes in the mevalonate pathway (Fig. 6A). The JH branch involves five enzymatic reactions (Fig. 6B; KEGG pathway map00981), and the first two (phosphatases and dehydrogenases) can be catalyzed by different enzymes from common biosynthetic pathways to produce farnesal; as a result, these enzymes have not been assigned to *Dp* in our analysis. The last two downstream steps, methylation (JHAMT) and epoxidation (CYP15A1), are considered the critical rate-limiting steps in the biosynthetic pathway [130] but can diverge between arthropod taxa, with the epoxidase-encoding gene *CYP15A1* missing in all noninsect species analyzed thus far [106]. Our study confirms the lack of *CYP15A1* in *Dp* (Fig. 6B), which, as proposed for other mites and crustaceans [33, 131], may indicate that methyl farnesoate, or even its precursor farnesoic acid, could be the bioactive sesquiterpenoid(s) of the species instead of JH III. The analysis of transcriptomic data revealed that no genes in the JH branch of the pathway were differentially expressed in a single stage/sex in all pairwise comparisons, but up to 4 genes in the mevalonate branch were significantly overexpressed in males (*HGMR* derpt03g03580, *MevK* derpt43g00340, *IPPI* derpt34g00190, and *FPPS* derpt36g00440) and one in females (*GPPPS* derpt13g00310). At this point, the implications of our results in the eventual sex-dependent regulation of sesquiterpenoid biosynthesis are unknown, since the mevalonate pathway is common in the biosynthesis of several other terpenoid secondary metabolites [135], such as monoterpenes, which will be described in Sect. " Biosynthesis of terpenoid pheromones".

#### Hormone receptors

As discussed for hormone biosynthetic enzymes, hormone receptors such as NRs are essential components of the endocrine signaling system. The screening of our genome for NRs (Additional file 2) identified potential orthologs of ecdysone NR (EcR-like, derpt23g00810) and ultraspiracle NR (USP-like, derpt06g00690). Both NRs have been shown to function as heterodimers that bind the 20E ecdysteoid and regulate molting in insect systems, and it is believed that they would function similarly in other arthropod lineages, such as crustaceans and chelicerates, where alternative ecdysteroid ligands are expected [106]. Indeed, the EcR ligand-binding domain in the spider mite *Panonychus citri* has been shown to structurally accommodate ponasterone A better than 20E [136]. In agreement with the putative role of these two NRs as ecdysteroid receptors during molting, they are expressed at significantly higher levels in *Dp* juveniles than in adults (for EcR-like) or females (USP-like). In the case of JH receptors, no orthologs were found for the known receptor partners identified in insects, the TFs *Met* (CG1705 in *Drosophila*) and *Tai* (CG13109) [106], indicating that other unknown receptors may fulfill analogous roles in *Dp*. Our knowledge of sesquiterpenoid pathways in Chelicerata remains elusive; on the one hand, *Met* appears to be absent in genomes such as those of the spider mite *T. urticae* [33], but it has been identified in some chelicerates, such as the spider *Parasteatoda tepidariorum* [131]. On the other hand, *Tai* has not been identified in any of these species and remains largely unexplored in this phylum. Since both TFs are members of the basic helix–loop–helix Per/Arnt/Sim (bHLH-PAS) family, we screened our predicted proteome and identified 6 bHLH-PAS-like proteins (derpt03g06460, derpt09g01060, derpt08g03740, derpt01g00660, derpt78g00010, derpt07g03230) bearing both the bHLH domain (IPR011598) and two PAS-like domains (annotated as IPR000014 and/or G3DSA:3.30.450.20). Notably, one of these genes, derpt07g03230, encoding a bHLH-PAS-like TF of low homology in databases (only in Sarcoptiformes), exhibited significantly higher expression in juveniles than in adults, which may be compatible with a hypothetical role in molting regulation as a sesquiterpenoid receptor.

#### Regulation of hormone biosynthesis and transport

A number of ecdysis-related neuropeptides potentially regulating sesquiterpenoid and ecdysteroid biosynthesis, as well as the initiation of molting behavior [106], were also identified in *Dp* (Additional file 2). We found genes encoding allatostatins (derpt04g06260, derpt04g06240) and their GPCR-like receptors (derpt34g00410, derpt19g00420, derpt36g00290), members of the CHH superfamily (derpt11g01690, derpt02g00140), insulin-like peptides (ILP; derpt42g00230, derpt05g02260, derpt08g04660), short neuropeptide F (sNPF; derpt03g05230), ETH (derpt03g364630) and its receptor ETHR (derpt04g94645), as well as bursicon alpha and beta subunits (derpt15g01380 and derpt15g01390, respectively) and a putative receptor (derpt01g10130). However, homologs of certain core components of this network previously identified in chelicerates were absent in *Dp* in our study (i.e. allatotropin, eclosion hormone, crustacean cardioactive peptides, and corazonin) [33, 106]. Notably, except for the ILP gene derpt42g00230, most of the identified genes did not show juvenile-enriched expression profiles in our analysis, as would be expected for molting-related genes. Some of these genes exhibited distinctive sex-biased expression, suggesting regulatory roles in processes other than ecdysis, particularly in reproduction. As introduced in Sect. "Other signaling factors", the two CHH-encoding genes presented opposite transcription profiles, one being male-associated (derpt02g00140; 137.3-fold overexpression compared with females) and the other being female-associated (derpt11g01690; 2.5-fold over males). In addition, both ETH and its receptor ETHR (derpt03g364630 and derpt04g94645, respectively) were overexpressed in adult females (5.3-fold over males, in both genes).

With respect to hormone transport, three genes encoding proteins bearing the hemolymph juvenile hormone binding domain (IPR010562) were identified in *Dp* (derpt12g00980, derpt26g00080, derpt19g00820). This domain is typically associated with juvenile hormone binding proteins (JHBPs) from insects, where they perform both transport and protection against degradation by esterases, thus contributing to the regulatation of JH titers in sensitive tissues. A similar function could be expected for these proteins in *Dp*, in this case by binding alternative non-JH III sesquiterpenoids, as recently shown in crustaceans [137]. Among these genes, derpt26g00080 presented substantially greater expression in immature stages than in females (> 16-fold), but not in males, indicating a possible role in ecdysis and/or reproduction.

#### Biosynthesis of terpenoid pheromones

Many of the aggregation, alarm and sexual pheromones described thus far in astigmatids are monoterpenes [1]. Notably, mite pheromones can be released in a stage/sex-specific manner in HDMs, as shown for the monoterpenes neryl formate or neryl propionate in *Dermatophagoides* [90]. As described above for sesquiterpenoids, the initial steps of monoterpene biosynthesis are included in the well-described mevalonate pathway. However, their downstream biosynthetic and signaling pathways remain poorly studied in most arthropods, including mites; thus, many of the genes involved still remain to be discovered [138, 139]*.* We identified 5 genes potentially involved in monoterpene conversion on the basis of the KEGG pathway map00907 (pinene, camphor and geraniol degradation): 4 oxidoreductases (derpt42g393850, derpt42g00060, derpt08g02600 and derpt11g01760) and 1 hydroxymethylglutaryl-CoA lyase (derpt02g08140). None of them presented a stage/sex-dependent transcription profile in our study. However, we found a potential ortholog of the functionally validated geraniol dehydrogenase from the astigmatid mite *Carpoglyphus lactis* (catalyzing the oxidation of geraniol to geranial) [140], which showed significantly greater expression in males (derpt22g00280; 4- to 46-fold higher than in other stages and females). Geranial has been previously detected as a volatile in *Dp* and an aggregation pheromone function has been suggested [141], although no experimental record is available.

### Cuticle remodeling

Arthropod growth and molting are strongly influenced by genes involved in cuticle formation, which are tightly regulated temporally and spatially [106, 142]. The cuticle´s exact composition, mechanical properties and permeability can vary greatly among different parts of the exoskeletal integument, different organs (e.g. pharynx, esophagus, hindgut, genitalia), and developmental stages [55, 143]. Here, we will explore genes that play a crucial role in the formation of the cuticle. These genes are specifically involved in the biosynthesis of chitin polymers and cuticle proteins (CPs).

#### Chitin metabolism

The balance between chitin degradation, resorption and biosynthesis required for cuticle remodeling during the molting cycle is maintained by the regulation of a number of enzymes. These include enzymes linked to chitinolytic pathways, such as chitinases (encoded by an expanded family of *n* = 16 genes in *Dp*), lytic polysaccharide monooxygenases (LPMOs; *n* = 2), N-acetylglucosaminidases (*n* = 4), chitin deacetylases (*n* = 11) and chitosanases (*n* = 2), and enzymes participating in the last steps of chitin biosynthesis from N-acetylglucosamine monomers, such as phospho-N-acetyl-glucosamine mutase (*n* = 1), UDP-N-acetyl-glucosamine pyrophosphorylase (*n* = 1) and chitin synthases (*n* = 3) (Additional file 2). Notably, in addition to cuticle formation, some of these genes might be associated with other processes, such as the digestion of dietary chitin (e.g. from ingested molds) or the formation of the gut´s peritrophic matrix, which also contains chitin fibrils.

Different stage/sex-dependent profiles were observed for these genes. While the most common profile among highly basally expressed chitinases corresponded to a significantly greater expression in nymphs than in females (derpt07g03760, derpt07g03780, derpt05g01070, derpt01g00950), other chitinases were overexpressed in juvenile stages (derpt03g06180, derpt05g01940), adults (derpt14g00960), or females (derpt18g01630). Additional chitinolytic genes, such as putative LPMOs, also presented divergent profiles across stages: derpt03g03050, which showed male-associated expression, and its contiguous gene in the genome, derpt03g03560, which was overexpressed in juvenile instars. Juvenile-overexpressed genes might be related not only to molting but also to dietary digestion (due to the increased feeding activity expected at these stages), whereas other genes may function in sexual development (e.g. female-associated chitinases). As commented for hormone biosynthesis genes, our sampling method did not include mites in the pharate stages under the active molting phase; thus, the full dynamics of the expression of these genes might not have been captured in our experiment.

#### Cuticular proteins

CPs are very diverse and comprise several families, as previously reviewed [49, 144]. With the aim of understanding how the expression of CP genes is regulated throughout juvenile and sexual development in *Dp*, we screened our assembly for known CP families previously described in insects [49, 55, 56, 143, 145] and, to a lesser extent in chelicerates [33–36, 146], together with a strategy to identify potentially new uncharacterized CPs (see methods for details on the screening strategies).

A total of 66 putative CP-encoding genes were identified among previously described CPs, and were classified into three families: 26 CPR-like (CP with chitin-binding Rebers and Riddiford, R&R, consensus domain), 1 CPLCP-like (CPs of low complexity and proline-rich), and 39 CPAP-like (CPs analogous to peritrophins) (Additional file 1: Table S4; Additional file 2). The selected CPAP-like candidates included proteins bearing at least one PF01607-like chitin-binding domain (CBD) but excluded chitinases or chitin deacetylases (in total 26, 6, 5, 1 and 1 encoded protein were identified containing 1, 2, 3, 4 or 7 CBDs, respectively; Additional file 1: Table S4). As a result of this approach, potentially true peritrophins from the gut’s peritrophic matrix were not distinguished from CPs. Both cuticular and peritrophin-like CBD-containing proteins are generally difficult to differentiate on the basis of sequence information, and in some cases, the same protein can be expressed both in the cuticle and in the gut [147]. Notably, Der p 23 (derpt10g00650) and Der p 37 (derpt02g02850), both CBD-containing proteins included in our list of CPAP-like proteins, have been shown to be immunolocalized in the *Dp* peritrophic matrix [148, 149].

The total number of CPs identified by this approach in *Dp* was relatively small compared with that found in other arthropod genomes, where CPs can commonly account for more than 1% of the total number of genes [144]. High CP abundance and diversity have been associated with the required plasticity of the cuticle properties in different tissues and developmental stages [55, 143]. While some CP families are widely distributed in insects, others can be restricted to only one insect order, such as apidermins in Hymenoptera [144]. This finding suggests that CPs are encoded by fast-evolving genes that are able to reach high specialization [143]. In addition, several hypothetical CPs have been identified in arthropod genomes that remain to be classified. In the case of Chelicerata, where fewer studies have been conducted [33–36, 146], the identified CPs have been restricted to a few families (CPRs and CPAPs). Therefore, it is very likely that novel chelicerate-specific CP families still need to be elucidated. For this reason, an *in-silico* strategy was adopted in this study to identify potentially new CPs in *Dp*, or “CP hypothetical” (CPH), following a previous designation [144]. In summary, CPH genes were selected when they met the following criteria: no potential function could be attributed (e.g. lacking protein domain annotations), high transcription level, bearing a signal peptide with predicted extracellular localization, and proteins exhibiting at least one sequence trait described for known CPs in the literature. Following this approach, a total of 2539 genes lacking functional predicted/manual annotations were initially identified, 238 of which presented very high expression in mite cultures (within the first decile of expression as per whole-transcriptome TPM values), and 70 were ultimately selected as CPHs on the basis of the presence of CP-related traits (Additional file 1: Table S5).

As described for previously characterized CPs, most of the identified CPHs in our study are relatively short proteins (> 70% were shorter than 300 aa), 89% of which lack cysteine residues in the mature peptide, and are predominantly composed of hydrophobic residues (> 90% of which with > 50% nonpolar residues in their sequence). These attributes, together with the presence of low complexity regions rich in amino acids such as glycine or proline, are expected to contribute to a general lack of protein structure, as can be observed when de novo prediction models are generated via Alphafold 2 [17] on selected CPHs (data not shown). The presence of disordered regions lacking secondary structures in CPs has been associated with their structural flexibility and high elasticity, which are often required for the cuticle [150]. A large group of 32 genes encoding homologs of the “*Dermatophagoides farinae* most abundant protein 2” (DFP2) [151] were identified as CPHs according to our criteria (homology group 1 in Additional file 1: Table S5). Their exact biological role remains unknown but has been recently associated with temperature stress responses in *D. farinae* [19]. Interestingly, the proteins encoded by two DFP2 genes, derpt02g02600 and derpt24g01500, present more than 10 copies of the short motif AAP(A/V/L), which, repeated three or more times in a single protein, is believed to be restricted to CPs [49]. Another identified group of CPHs showed a high density of PV and PY pairs, a typical trait defining known CPLCPs [55, 145] and the CPLCP-like *Dp* gene derpt04g06580. Among this group, we identified derpt04g06550, which is adjacent in the genome and a homolog of derpt04g06580 (52% protein sequence identity), as well as derpt01g291950, which encodes a homolog of the *Drosophila* CPLCP Vajk4 (CG30101).

TPM-based heatmap analysis of the expression of CPR-like, CPAP-like and CPH groups (136 genes in total) indicated that most of the genes presented higher expression in juvenile stages than in adults, particularly in larvae (Additional file 1: Fig. S5). Significant differential expression was recorded for some of the genes with the highest expression in each group in pairwise comparisons between the juvenile and adult stages (i.e. CPR derpt10g01430 and derpt01g01670; CPAP derpt10g00640; CPHs derpt02g02600, derpt13g01770, derpt09g02750 and derpt24g01270) (Additional file 2). These results are consistent with the high demand for cuticle synthesis/remodeling during the molting and growth phases in larvae and nymphs [34, 35]. In contrast, a lower proportion of genes presented a clear sex-enriched expression profile. Most of the significantly overexpressed genes with this profile corresponded to CPHs, particularly derpt04g93805 and derpt04g01290 in males, and derpt44g00350, derpt07g04060, derpt44g00370, derpt16g02280 and derpt17g01100 in females, whose expression levels were very high (TPM > 3,500) and > 20-fold greater than those in the other adult sex. In addition, CPAP derpt16g02560 was significantly overexpressed in females. The transcription profiles of these sex-specific genes may indicate some degree of specialization in adult-specific cuticles, such as those lining reproductive organs or those conforming the chorion of eggs within gravid females.

### Allergen-related proteins

**Fig. 7:**
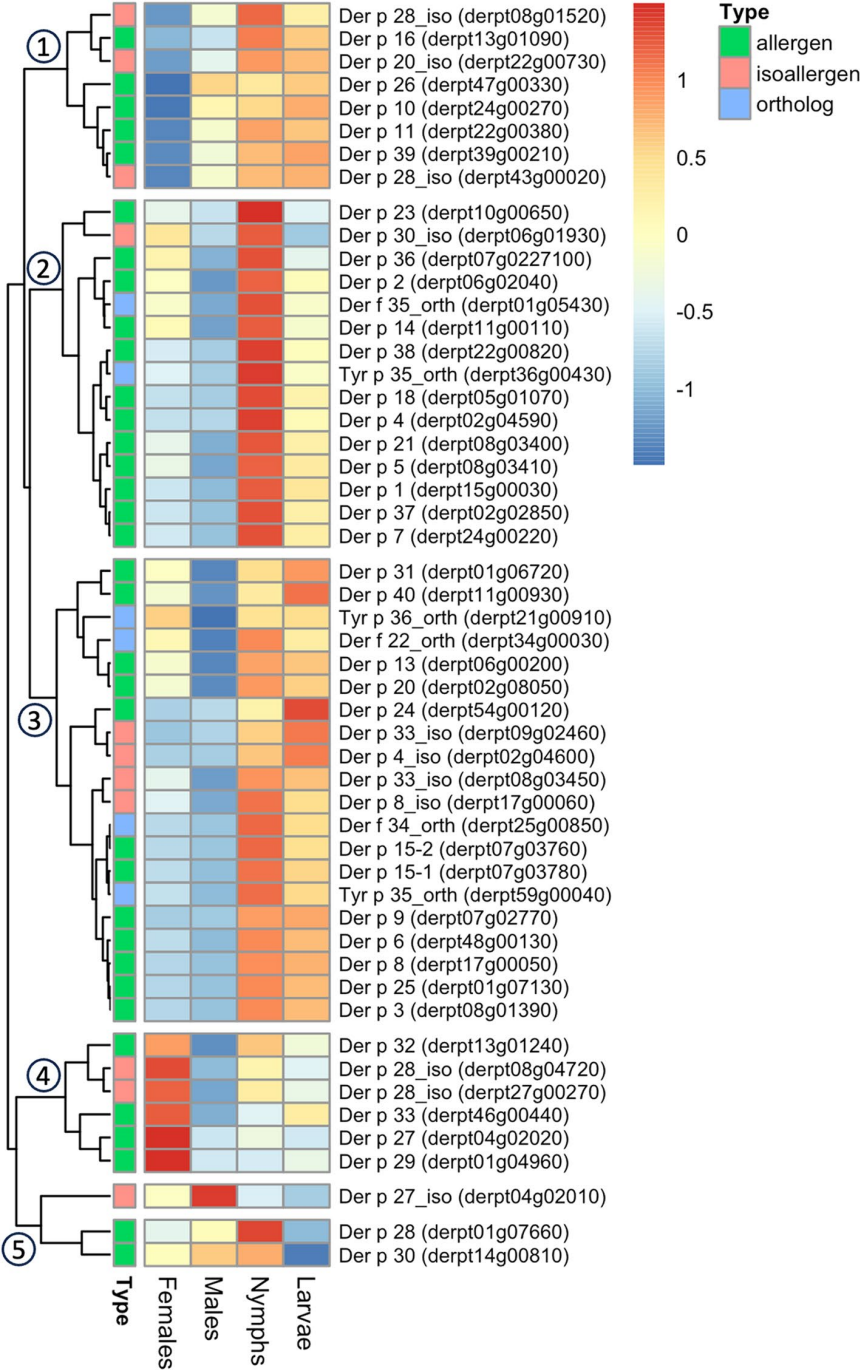
Stage-specific expression heatmap of allergen-related genes in *D. pteronyssinus*. The darker red color indicates a higher transcript per million average (from n = 3 replicated libraries). Colored boxes next to the gene clustering dendrogram indicate the type of allergen-related gene, as indicated in the legend: officially recognized allergens [3]; putative isoallergens; putative orthologs of known allergens from other house dust mite species. Allergen-related names are indicated in each plot together with gene ID (in brackets). Numbers located at branch roots indicate different gene clusters

Stage and sex-dependent transcription profiles of genes encoding the 34 currently recognized allergens [3], 11 putative isoallergens, and 6 orthologs (of known allergens from other HDMs) previously annotated in our *Dp* assembly [29] (Additional file 2) will be described here. As shown in the cited study, the reported basal expression of these genes in our samples was generally very high (up to 22,095.4 TPM units for Der p 1 in nymphs), with the exceptions of the allergen Der p 29 (maximum TPM at any stage/sex = 54.4) and the putative isoallergens Der p 4-like, Der p 28-like (derpt43g00020) and Der p 27-like (5.2, 29.9 and 74.1 maximum TPM, respectively). Clustering heatmap analysis comparing the transcription profiles of these genes revealed that the most common profile corresponded to a greater expression in immature stages than in adults (Fig. 7, clusters 2 and 3), although only the Der p 33 isoallergen derpt09g02460 presented significantly greater expression in juveniles than in adults in all pairwise statistical comparisons (Additional file 2). These apparently divergent results are a consequence of the twofold change cutoff established in our pairwise analysis. When only the FDR-adjusted *p* value ≤ 0.05 criterion is used (at any fold change), 32 allergen and allergen-related genes are significantly overexpressed in pairwise comparisons between nymphs and adults of both sexes (including allergens Der p 1, 2, 3, 4, 5, 6, 7, 8, 9, 4, 15_1, 15_2, 16, 18, 20, 21, 23, 25, 31, 36, 37 and 38; data not shown). Other transcription profiles, such as over- and underexpression in females, were also recorded and are described and discussed below for specific gene groups. The following subsections are organized according to groups of functionally and/or structurally associated allergens and allergen-related proteins. In addition, for some of these allergens, genes belonging to the same family or showing similar functions are also discussed in the corresponding subsections.

#### Muscle and cytoskeleton-related

Eight of the allergen-related genes identified in *Dp* encode cytoskeletal proteins potentially related to the development and function of muscle tissues (Additional file 2). Notably, 5 of these genes presented significantly lower transcription in adult females than in other stages; namely, Der p 10 (tropomyosin; derpt24g00270), Der p 11 (paramyosin; derpt22g00380), Der p 16 (gelsolin; derpt13g01090), Der p 26 (myosin light chain; derpt47g00330) and Der p 39 (troponin C; derpt39g00210) (all included in cluster 1 of Fig. 7). Similarly, presenting the same profile, we also identified the Der p 20 isoallergen gene derpt22g00730, encoding an AK involved in energy metabolism that is also expected to be abundant in muscle tissues (see the discussion at the end of Sect. "Other proteins related to male reproductive biology"). In addition, the Der p 33 isoallergen gene derpt09g02460, encoding an alpha-tubulin, was significantly overexpressed in juveniles compared with adults.

The high frequency of genes underexpressed in females in this group is consistent with our functional enrichment analysis results for the network gene cluster “female-reduced expression” (Fig. 2, cluster 7), which revealed enrichment of the GO term “actin cytoskeleton organization” (GO:0030036) (Sect. "Network analysis and functional annotation of stage-specific gene groups"). To broaden the scope of our analysis, of the 144 genes annotated in the genome as being associated with the cytoskeleton (using primarily the KEGG pathways map04820 and map04814 as references; Additional file 2), 36 showed significant repression in adult females according to all pairwise comparisons. In addition to the allergen genes cited above, female-repressed genes were included in families such as troponins (4 out of 4 annotated genes), calponins (3/5), filamins (4/5), myosin regulatory light chain 9 proteins (2/3), LIM domain cysteine and glycine-rich proteins (4/6), and cyclase-associated CAP proteins (2/2). Our observations are in line with previous results reported on the mite *P. ovis* [34] indicating possible differences in the muscular anatomy of adult females. These differences could be explained by the expected reduced motility/locomotion activity of female mites compared with that of adult males (e.g. when seeking for mating encounters) or actively feeding juvenile stages (e.g. when seeking for food). Finally, as described in Sect. "Genes with a known role in oogenesis", a number of other cytoskeletal-related genes not associated with muscle functions but rather with the formation of the mitotic/meiotic microtubule spindle were significantly overexpressed in females, none of which encode known allergens.

#### ML domain-containing

Lipid-binding proteins containing the myeloid differentiation factor-2-related lipid recognition (ML) domain comprise at least three different allergen groups in HDMs: the major allergen group 2 (Der p 2, derpt06g02040), group 22, and group 35. To date, the allergenicity of the latter two groups has been confirmed only in *D. farinae* [3], but orthologs can be found in *Dp* (Der f 2-like derpt34g00030 and Der f 35-like derpt01g05430, respectively)*.* Notably, as suggested for other lipid-binding allergens from different origins, their ability to bind lipid cargos can further promote allergenicity by mediating interactions with human innate immunity receptors [152–154]. In addition to the three cited genes, 5 other genes encoding ML proteins of unknown allergenicity were identified in *Dp* (derpt06g03660, derpt11g01450, derpt01g03210, derpt02g05090, derpt15g00330). The transcriptomic analysis of this gene family (n = 8) revealed different expression patterns indicative of potentially distinct biological roles in mites.

First, the three allergen (or allergen ortholog) genes presented very high overall expression (up to 19,411.0, 623.2 and 8,698.9 TPM units for Der p 2, Der f 22-like and Der f 35-like, respectively) but no significant differences between stages/sexes (Additional file 2), indicating putative basal functions. The three allergen groups have been detected by proteomics in mite fecal products [155–157], pointing to an association with the digestive physiology of the mite. Moreover, group 2 allergens have been immunolocalized in the mite gut [158, 159]. An immunological function has been previously suggested for these proteins on the basis of their resemblance to mammalian homolog proteins such as MD-2 [154]. Specifically, by binding liposaccharide endotoxins (LPS) from bacteria, which are rich in HDM guts [160, 161]. However, a recent work supports an alternative function for at least Der p 2, which appears to have a low affinity for bacterial LPS, with cholesterol being its putative natural ligand [152]. Cholesterol is essential for many developmental processes, including the biosynthesis of ecdysteroid hormones, but, as shown in other arthropods, mites cannot synthesize it de novo and need to acquire it from dietary sources [1, 106, 135]. Taken together, this information (high basal expression across stages, gut localization, and cholesterol binding) could suggest a hypothetical digestive function for these proteins, consisting in the sequestration of dietary cholesterol prior to its absorption in the mite gut. Evidence of such a role is lacking and would justify future experimental studies.

Among the remaining nonallergen ML-related genes, three presented moderate expression and a significant sex-dependent profile, suggesting specialized roles: two were notably enriched in females (derpt06g03660 and derpt01g03210; 3.5- and 3.7-fold greater than in males, respectively) and one was enriched in males (derpt11g01450; 50-fold greater than in females). The high sequence variability among *Dp* ML proteins (pairwise identities below 40%) suggests diverse affinities for small lipid ligands in vivo, which, on the basis of the sex-specific expression of the cited genes, could be related to lipids, such as hormones, that participate in reproduction (see Sect. "Hormone and pheromone metabolism, signaling and transport"). In support of this hypothesis, a recent study on the hemipteran insect *Nilaparvata lugens* has demonstrated that the ML family of proteins can regulate lipid metabolism, including the biosynthesis of ecdysteroid hormones, and, in some cases, be associated with the male testis [162]. Similarly, predominant expression of ML protein-encoding genes has also been recorded in spermatophores of the tick *Dermacentor variabilis* [66] and in males of the poultry red mite *D. gallinae* [35].

#### Group 7-related

Der p 7 and Der f 7 are considered mid-potency allergens based on their allergenicity toward allergic patients [4]. In *Dp*, in addition to Der p 7 (derpt24g00220), 9 other genes encoding proteins with the so-called “group 7 domain” (IPR038602) were found (derpt48g00100, derpt02g05580, derpt48g00110, derpt12g00980, derpt01g03870, derpt01g05250, derpt02g05570, derpt48g00120, derpt48g088075). The potential allergenicity of these other proteins is currently unknown, as is the possible molecular function of the shared protein domain, except that it may bind lipids. The lipophilic properties of group 7 allergens have been inferred from their distant structural similarity to bactericidal/permeability-increasing proteins, as well as from a discrete number of binding assays [153, 163]. Notably, in *Dp*, the group 7 domain could be associated with a second lipid-binding domain, the JHBD domain (IPR010562; extended information in Sect. " Regulation of hormone biosynthesis and transport"), as observed in the product of derpt12g00980. Proteins with these two domains have also been found in the crustacean species *Scylla paramamosain* [137], where a role in immunity rather than sesquiterpenoid hormone binding has been suggested. Other studies have shown affinity of group 7 allergens for a bacterially derived lipid product (polymyxin B) but not for JH III or methoprene (a JH analog) [153, 163]. Our transcriptomic analysis revealed that the 2 genes most highly expressed in this group (Der p 7 and derpt01g03870; TPM values up to 5,676.3 and 4,383.3, respectively) were not differentially regulated across stages/sexes, whereas 3 genes (derpt48g00110, derpt48g088075, derpt48g00120) presented notably increased expression in females (14.0-, 20.3- and 13.4-fold greater than in males, respectively) (Additional file 2). An immune function could be compatible with these female-associated genes since, as shown in *Drosophila*, immune defense genes can be induced to prevent bacterial infections in females after mating [164].

#### Groups 5 and 21

A third class of lipid-binding allergens of clinically relevant allergenicity but unclear biological function are homologous proteins of groups 5 and 21, both bearing the mite-specific protein domain IPR020306 [4]. Despite having only 33% sequence identity, these allergens share a similar three-helical-bundle structure, which denotes lipophilic properties [165–167]. In our study, Der p 5 (derpt08g03410) and Der p 21 (derpt08g03400), which are contiguous in the genome, presented similar transcription patterns (Fig. 7). Previous transcriptomic results from our colony assessing the impact of factors such as temperature, relative humidity or diet composition were also in agreement with a similar transcriptional regulation of these genes [8, 29, 168]. Interestingly, we showed that the expression of Der p 5, Der p 21 and derpt01g03820 (which share the same domain) was induced after the ingestion of the peptidase inhibitor cystatin A, which might relate these proteins with the digestive physiology of the mite [29].

#### Peptidase and peptidase inhibitor allergens

Five of the known *Dp* allergens are proteolytic enzymes, namely, the major allergen and cysteine endopeptidase Der p 1 (derpt15g00030), serine endopeptidases Der p 3, 6 and 9 (derpt08g01390, derpt48g00130 and derpt07g02770, respectively), and the NLPC/P60 domain endopeptidase-like Der p 38 (derpt22g00820). The expression profile identified by clustering heatmap analysis for all these peptidase allergen genes corresponds to a greater transcription in immature stages than in adults (Additional file 1: Fig. S6, cluster 3; see annotations for allergens in the legend). This profile, together with the high basal expression of these peptidase allergens (Additional file 2), is consistent with a potential feeding/digestive role, as previously proposed in the sheep scab mite *P. ovis* for Pso o 1 (ortholog of Der p 1), which was upregulated upon feeding and overexpressed in tritonymphs [34]. Juvenile-stage mites are expected to show higher feeding activity than adult mites because of their increased growth rate, as it occurs in insects [169]. In agreement with these findings, the functional enrichment analysis conducted in our study revealed that molecular functions related to energy generation were enriched in juveniles (Sect. "Network analysis and functional annotation of stage-specific gene groups"). Cysteine endopeptidases, including Der p 1, are known to be the most hydrolytically active peptidases in *Dp* mite body extracts, followed by serine endopeptidases, which are mostly active in fecal pellets [5]. Notably, 10 cysteine endopeptidase genes other than Der p 1 also presented a similar immature-enriched transcription profile in our study (Additional file 1: Fig. S6, cluster 3; see annotations for enzyme families in the legend), including the highly expressed papain-like genes derpt05g03150, derpt05g03160 and derpt21g00090 (up to 4,332.4, 2,305.3 and 293.7 TPM units, respectively). These 3 peptidases have been recently identified as potential key players in the proteolytic digestion of *Dp* on the basis of their induction after cystatin A ingestion [29].

Two allergen-related genes encoding serpin-like peptidase inhibitors, the Der p 27 allergen derpt04g02020 and its putative isoallergen derpt04g02010, presented opposed differential expression profiles, being biased towards female and male adult sexes, respectively (Fig. 7; Additional file 2). As discussed in previous sections, the regulation of serpin genes may respond to specific roles of this family in either male or female reproductive processes.

### Horizontally transferred genes

Recent studies have demonstrated that HGT from microbial donors is a common event in mite genomes, including those of HDMs [24, 138, 170–172]. Herein, we searched our annotated genome for previously HGT genes identified in mites according to the literature and extended this list by conducting a mining approach based on the *h*-index metric to detect new HGT candidates in our assembly [57] (see Methods section). A total of 48 putative HGT genes were detected in *Dp*, 7 of which were identified in this study (Additional file 1: Table S6; Additional file 2). The most common functional category among HGT genes corresponds to detoxification (with an expansion in the UDP glucuronosyltransferase family, UGT), followed by lysis of fungal and bacterial cell walls. The less represented categories included metabolism of sugars, amino acids, and nucleic acids; DNA repair; and terpene biosynthesis (Additional file 1: Table S6). Our results are consistent with previous evidence in other arthropods, where the acquisition of microbial enzyme-encoding genes can confer new adaptive traits and metabolic capabilities to the recipient organism [138, 170, 172, 173].

Heatmap clustering analysis revealed that the most abundant transcription profile among these genes consisted of an increased expression in juvenile stages (Additional file 1: Fig. S7, cluster 3). Notably, most of the HGT genes that have been functionally classified in the lysis of microbial cell walls group (Additional file 1: Table S6) were included in heatmap cluster 3 (Additional file 1: Fig. S7; predominant in juveniles), which, as discussed in the previous section for peptidases, would be compatible with a potential role in digestion. A similar result has been reported for cell wall-degrading enzymes in nymphal stages of the oribatid mite *Archegozetes longisetosus* [138], where the presence of HGT genes encoding lytic enzymes was attributed to its detritivorous feeding habit in the soil. Indeed, it is believed that Astigmata, including HDMs, may have diverged from Oribatida soil mites [24]. HDMs maintain the ability to feed on microbes as part of their diet, especially filamentous fungi in the case of *Dp* [31]. Among the significant DEGs that were overexpressed in juveniles compared with adults, three genes encoding potential lytic enzymes of the fungal cell walls, including one chitinase (derpt05g01940) and two beta-1,3 glucanase genes (derpt02g00800, derpt02g00810) (up to 3.7-, 3.4- and 4.6-fold overexpression, respectively; Additional file 2), were detected. Among the genes included in heatmap cluster 4 (Additional file 1: Fig. S7; predominant in females), another glucanase gene (derpt22g00810) was significantly overexpressed in females (up to 7.9-fold), which suggests alternative roles. In addition, derpt12g01170, encoding a DNA photolyase that may play a role in DNA repair after UV damage, was also overexpressed in females compared with immature instars (up to 2.6-fold). Although not being identified in previous analyses, the result for this gene is consistent with the overall induction of DNA repair and mitosis/meiosis-associated genes in females discussed in previous sections. Finally, derpt14g00520, encoding a terpene synthase, was overexpressed in males (up to 6.3-fold; Additional file 1: Fig. S7, cluster 2). Homologous terpene synthase genes have been found in other astigmatid mites [24], and 17 such HGT genes have been detected in the trombidiid mite *L. delicense* [170]. The secondary metabolites synthesized in *Dp* by this enzyme are still unknown. Among other functions, the male-associated expression of this gene could be compatible with the synthesis of sex-dependent terpenoid pheromones, the biosynthetic pathways of which remain to be explored (see Sect. " Biosynthesis of terpenoid pheromones").

## Conclusions

Herein, we present the first transcriptomic survey on gene expression across developmental stages and adult sexes of a HDM species. From whole-transcriptome network and functional analyses to the study of specific gene families, we provide insight into particular aspects of the *Dp* biology. Our primary focus has been on biological processes that are directly dependent on the developmental stage of the mite, such as distinct facets of male and female reproduction, or the endocrine control and cuticle remodeling processes associated with ecdysis. In addition, by studying allergen-related and HGT genes, we address other important areas of mite biology, such as digestion, the cytoskeleton and mite-microbe interactions.

The identification of key *Dp* genes underlying many of these essential biological processes and the study of their regulation across stages can be used as a baseline to select candidate targets for the future development of new biorational management methods for *Dp* and other noxious mite species. Different strategies are currently emerging that exploit genomic resources and provide unprecedented opportunities in this area of research, including gene silencing by dsRNA [11, 12, 20, 21], the discovery of new bioactive molecules from compound libraries via molecular docking [15, 16], or the design of toxic proteins de novo via deep learning-based methods [174]. Among possible approaches, the disruption of reproduction (e.g. vitellogenin and oogenesis regulator genes) and ecdysis (e.g. hormone biosynthesis/regulation or cuticle formation) have been key focus areas in pest management research [12, 104, 175]. Other gene candidates could be linked to processes such as energy metabolism (e.g. AK) [176] or the nervous system (e.g. acetylcholinesterase derpt06g02510).

Our thorough study also revealed a number of genes and gene families with characteristic stage-dependent transcription profiles but still of unknow function, which merits further research. These genes generally show poor homology to genes of other non-mite species in databases, and some of them have been described for the first time in this report and/or show family expansions. Examples of such genes, organized by their transcription profile, include i) male-associated TspL-, AMP- and 4BetaP-like genes; ii) female-associated odorant-binding-like genes or highly expressed metallocarboxypeptidase derpt07g01560; and iii) CPH-like genes of different stage/sex-specific profiles, some of which have the top basal transcription levels in our study.

Stage/sex-dependent expression was demonstrated for a number of known *Dp* allergen genes, suggesting potential effects of mite population structure on the profile of allergens produced, for example, by large-scale pharmaceutical cultures (therefore, having a potential impact on the standardization of diagnosis and immunotherapy allergenic extracts). In addition, the generated information on allergen expression contributes to improving our understanding of their regulation and potential physiological function, which for many of these proteins remains elusive. One of the most common trends detected by heatmap analysis among allergen-encoding genes corresponded to increased transcription in juvenile stages, particularly in nymphs, which relates many of these genes with the active growth and digestion status associated with immature developmental stages (as also observed for many nonallergen proteases). These results are in line with previously documented results comparing the gene expression of selected allergens by RT-qPCR between larvae, nymphs and mixed adults or between both adult sexes [8]. Other allergens of known function related to the cytoskeleton organization were expressed at significantly lower levels in females compared with other stages, indicating differences in the muscular anatomy of females. This was also observed for many other nonallergen cytoskeletal genes. In addition, by studying the expression of clinically relevant yet functionally unknown lipid-binding allergens (Der p 2, 7, 5, and 21) and other genes within their families, we propose case-specific hypotheses linking them to the mite’s digestive physiology, immune system, or interactions with lipid-based reproductive hormones (or their precursors). Future mechanistic studies will be essential to further elucidate the specific roles of these allergens.

In summary, the extensive annotation and in-depth transcriptomic analysis conducted in this report, together with the comprehensive dataset being released in combination with the interactive and publicly available ORCAE platform, offer a valuable resource for future investigations into the biology of *Dp*, HDM and Astigmata mites in general.

## Supplementary Information

Additional file 1. Supplementary figures and tables. This document includes supplementary figures S1, S2, S3, S4, S5, S6 and S7; and supplementary tables S1, S2, S3, S4, S5 and S6.

.Additional file 2. Stage-dependent expression and annotation data for the whole *Dermatophagoides pteronyssinus* transcriptome. The spreadsheet is organized in colored panels, from left-to-right: gene reference data in blue (locus ID in ORCAE, description and short name, encoded protein length); RNAseq read count data in green (TPM values and network analysis clustering); pairwise differential expression data in white (fold change and adjusted P-value for each comparison between stages; F, M, N and L denoting females, males, nymphs and larvae, respectively); database-derived annotation data in yellow (protein domains, GO terms, KEGG onthology, OrthoMCL groups, *D. melanogaster* BLASTp homologs); and, in green, assignment to functional groups and categories described in the main text (in this order: Peptidases, Peptidase inhibitors, Immunity, Cell adhesion and epithelial barrier, Oxidoreductases, Lipases/esterases, Glycosylases, Protein kinases, Transcription factors, Nuclear Receptors, Cytokines and neuropeptides, G Protein-Coupled receptors and cytokine receptors, Small GTPases and associated proteins, Oogenesis-related, Hormone biosynthesis pathways, Chitin metabolism, Cuticular Proteins, Allergen-related proteins, Cytoskeleton-related, and Putative Horizontally Transferred Genes). Each column can be filtered at the user ´s convenience using Microsoft Excel or similar packages.

Additional file 3. Significantly enriched GO terms for selected network analysis gene clusters. Each tab in the spreadsheet refers to an independent analysis and is coded as follows: BP or MF, which denote either “Biological Process” or “Molecular Function” terms, followed by the network gene cluster name under study (“Female_UP” refers to “cluster 1” of Figure 2 in the main text; “Male_UP” to “cluster 2”; “Juvenile_UP” to “cluster 6”; “Female_DOWN” to “cluster 7”; “Male_DOWN” to “cluster 8”; “Adult_UP” to “cluster 5”).

Additional file 4. Significantly enriched KEGG pathways for selected network analysis gene clusters. Each tab in the spreadsheet refers to the analysis of a single network gene cluster, which is designated as follows: “Female_UP” refers to “cluster 1” of Figure 2 in the main text; “Male_UP” to “cluster 2”; “Juvenile_UP” to “cluster 6”; “Female_DOWN” to “cluster 7”; “Male_DOWN” to “cluster 8”; “Adult_UP” to “cluster 5”.

## Data Availability

The data generated or analyzed during this study are included in this published article (and its supplementary information files) as well as public repositories. RNAseq raw data supporting the findings of this study are available in the NCBI Bioproject ID PRJNA1233935 (https://www.ncbi.nlm.nih.gov/bioproject/PRJNA1233935). Genomic information is available via the NCBI Bioproject ID PRJNA843460 (https://www.ncbi.nlm.nih.gov/bioproject/PRJNA843460). In addition, all the analytical information generated for the complete transcriptome in this study is available in the supplementary “additional file 2”. Any unavailable data will be accessible upon reasonable request to the corresponding authors.

## Abbreviations

HDM: House dust mite
Dp: *Dermatophagoides pteronyssinus*
ORCAE: Online Resource for Community Annotation of Eukaryotes
TPM: Transcripts per million
PCA: Principal component analysis
GO: Gene Ontology
KEGG: Kyoto Encyclopedia of Genes and Genomes
KO: KEGG Ontology
HGT: Horizontal gene transfer
CP: Cuticular protein
PDB: Protein Data Bank
FDR: False discovery rate
DEG: Differentially expressed gene
BP: Biological process
MF: Molecular function
SFP: Seminal fluid proteins
AMP: Antimicrobial peptide
Tsp: Tetraspanin
TspL: Tetraspanin-like protein
SORD: Sorbitol dehydrogenase
CYP: Cytochrome P450
PK: Protein kinase
TSSK: Testis-specific serine kinase
TF: Transcription factor
NR: Nuclear receptor
GPCR: G protein-coupled receptor
AK: Arginine kinase
4BetaP: Male-specific four-bladed beta-propeller fold family
CHH: Crustacean hyperglycemic hormone
ETH: Ecdysis triggering hormone
JH: Juvenile hormone
EcR: Ecdyson nuclear receptor
USP: Ultraspiracle nuclear receptor
ILP: Insulin-like peptide
sNPF: Short neuropeptide F
JHBP: Juvenile hormone binding protein
LPMO: Lytic polysaccharide monooxygenase
CPR: Cuticular protein with chitin-binding Rebers and Riddiford domain
CPLCP: Cuticular protein of low complexity and proline-rich
CPAP: Cuticular protein analogous to peritrophins
CBD: Chitin-binding domain
CPH: Cuticular protein hypothetical
DFP2: *Dermatophagoides farinae* Most abundant protein 2
ML: Myeloid differentiation factor-2-related lipid recognition
LPS: Liposaccharide endotoxin
UGT: UDP glucuronosyltransferase family
