# Supplementary material for: Stage-specific transcriptomic analysis reveals insights into the development, reproduction and biological function of allergens in the European house dust mite Dermatophagoides pteronyssinus
Source: BMC Genomics. 2025 May 26;26:527. doi: 10.1186/s12864-025-11703-w (PMC12105342; doi:10.1186/s12864-025-11703-w)
Supplement: Supplementary file 1 — Additional file 1. Supplementary figures and tables. This document includes supplementary figures S1, S2, S3, S4, S5, S6 and S7; and supplementary tables S1, S2, S3, S4, S5 and S6. [file 12864_2025_11703_MOESM1_ESM.pdf]

SUPPLEMENTARY INFORMATION – Additional file 1

Stage-specific transcriptomic analysis reveals insights into the development, reproduction and biological function of allergens in the European house dust mite *Dermatophagoides pteronyssinus*

José Cristian Vidal-Quist, Félix Ortego, Bart N. Lambrecht, Stephane Rombauts, and Pedro Hernández-Crespo

SUPPLEMENTARY FIGURES:

Figure S1. Overlap of network analysis gene clusters with the "DNA replication" reference KEGG pathway map (map03030). Colored boxes indicate components of the pathway supported by genes identified in the *D. pteronyssinus* genome; each color denotes a gene cluster (or group of clusters) as indicated at the legend below. White boxes indicate components not supported by any annotated gene in the *D. pteronyssinus* genome. Results and original image file were obtained using the KEGG Mapper Color tool ([1]; Kanehisa Laboratories) using a list of KEGG Ontology (KO) terms annotated from the *D. pteronyssinus* proteome and marked according the clustering results.

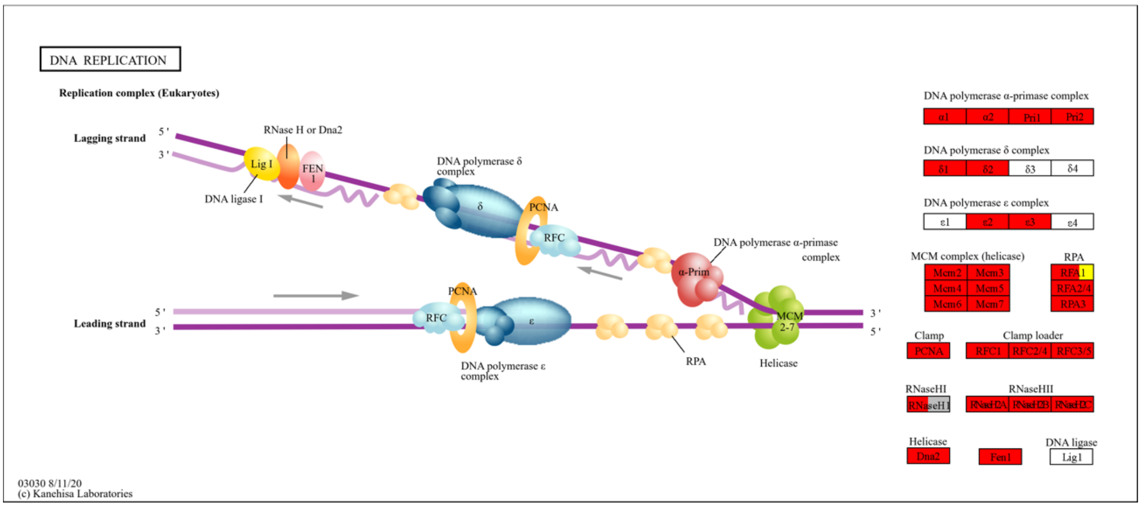

Cluster ID	Network analysis expression profile	Color
1	Adult females (F)	
2	Adult males (M)	
5	F-M (Adults)	
6	N-L (Juveniles)	
3,4,7-14	Other profiles	

Figure S2. Overlap of network analysis gene clusters with the "Nucleotide excision repair" reference KEGG pathway map (map03420). Colored boxes indicate components of the pathway supported by genes identified in the *D. pteronyssinus* genome; each color denotes a gene cluster (or group of clusters) as indicated at the legend to the right. White boxes indicate components not supported by any annotated gene in the *D. pteronyssinus* genome. Results and original image file were obtained using the KEGG Mapper Color tool ([1]; Kanehisa Laboratories) using a list of KEGG Ontology (KO) terms annotated from the *D. pteronyssinus* proteome and marked according the clustering results.

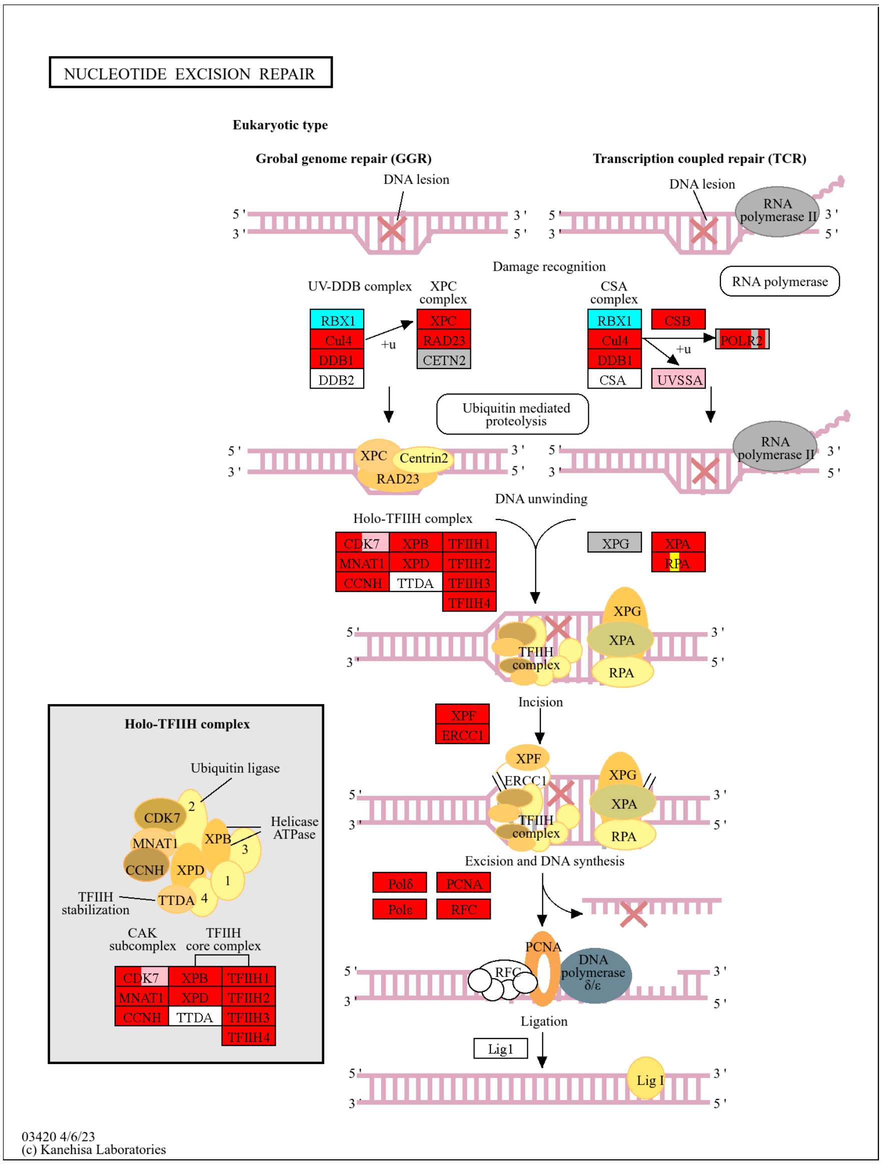

Cluster ID	Network analysis expression profile	Color
1	Adult females (F)	
2	Adult males (M)	
5	F-M (Adults)	
6	N-L (Juveniles)	
3,4,7-14	Other profiles	

Figure S3. Overlap of network analysis gene clusters with the "Nucleocytoplasmic transport" reference KEGG pathway map (map03013). Colored boxes indicate components of the pathway supported by genes identified in the *D. pteronyssinus* genome; each color denotes a gene cluster (or group of clusters) as indicated at the legend below. White boxes indicate components not supported by any annotated gene in the *D. pteronyssinus* genome. Results and original image file were obtained using the KEGG Mapper Color tool ([1]; Kanehisa Laboratories) using a list of KEGG Ontology (KO) terms annotated from the *D. pteronyssinus* proteome and marked according the clustering results.

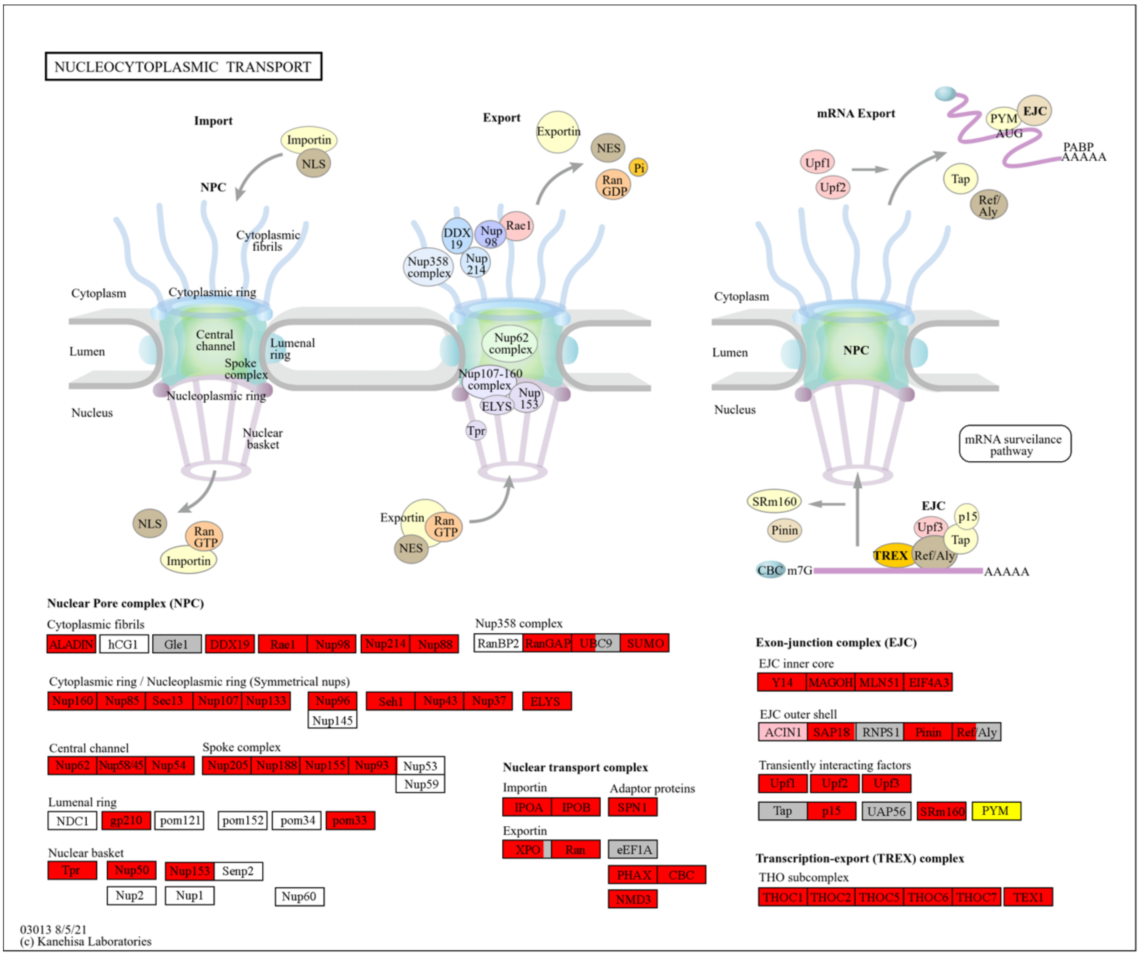

Cluster ID	Network analysis expression profile	Color
1	Adult females (F)	
2	Adult males (M)	
5	F-M (Adults)	
6	N-L (Juveniles)	
3,4,7-14	Other profiles	

Figure S4. Structural features of undescribed "Male specific four-bladed beta-propeller fold" (4BetaP) family proteins. Panel A) Structure prediction model for derpt27g00030 (LOC113794088, AlphaFold Protein Structure Database). The C-terminal unstructured end of the protein (105 amino acids) has not been depicted for visualization purposes. Panel B) Manually curated structure-based sequence alignment of three 4BetaP-like proteins (derpt27g00030 with 3 predicted disulfide bonds, derpt03g00530 with 2 disulfide bonds, and derpt56g00410 with 1 disulfide bond). Only the sequence corresponding to the four-bladed beta-propeller fold is shown. Pink annotations denote alpha-helixes, yellow arrows indicate beta-strands, and the cysteines forming disulfide bonds are marked in yellow. Four beta-strand rich regions can be observed per protein, which correspond to each of the for blades depicted in panel A.

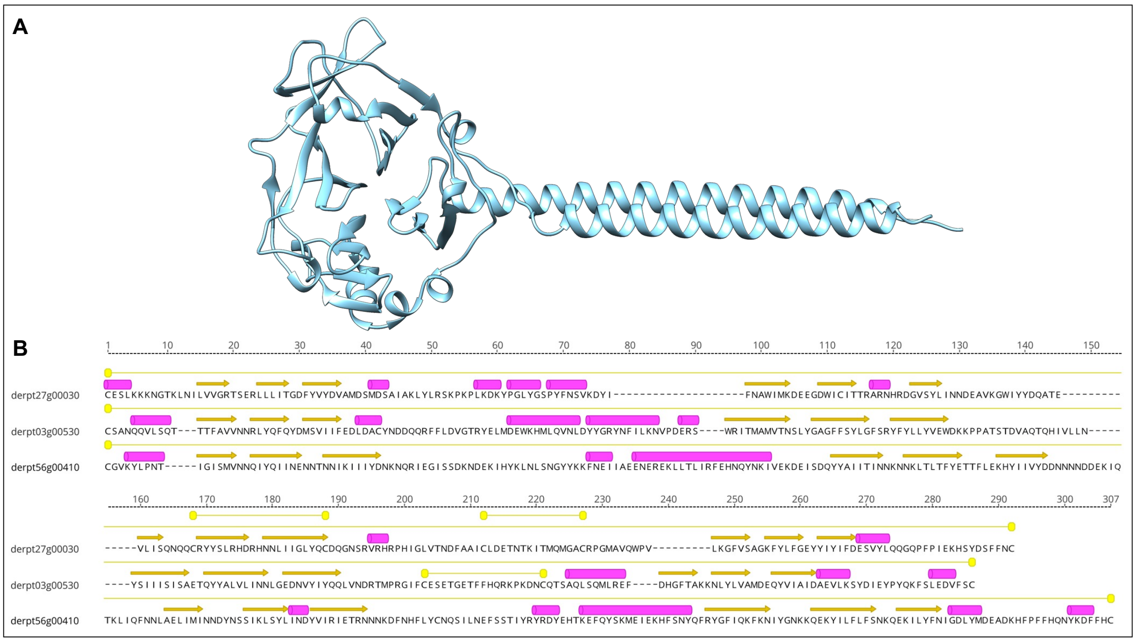

Figure S5. Stage-specific expression heatmap of cuticular proteins (CP) in *D. pteronyssinus*. The darker red color indicates a higher transcript per million (TPM) average (n = 3 replicated libraries). Panel A) Expression level across stages of CPR-like (CP with chitin-binding R&R consensus) genes (n = 26). Panel B) Expression level across stages of CPAP-like (CPs analogous to peritrophins) genes (n = 39). Panel C) Expression level across stages of CPH-like (CP hypothetical) genes (n = 70 + derpt04g06580 CPLCP, CPs of low complexity and proline-rich). Gene IDs are indicated in each plot.

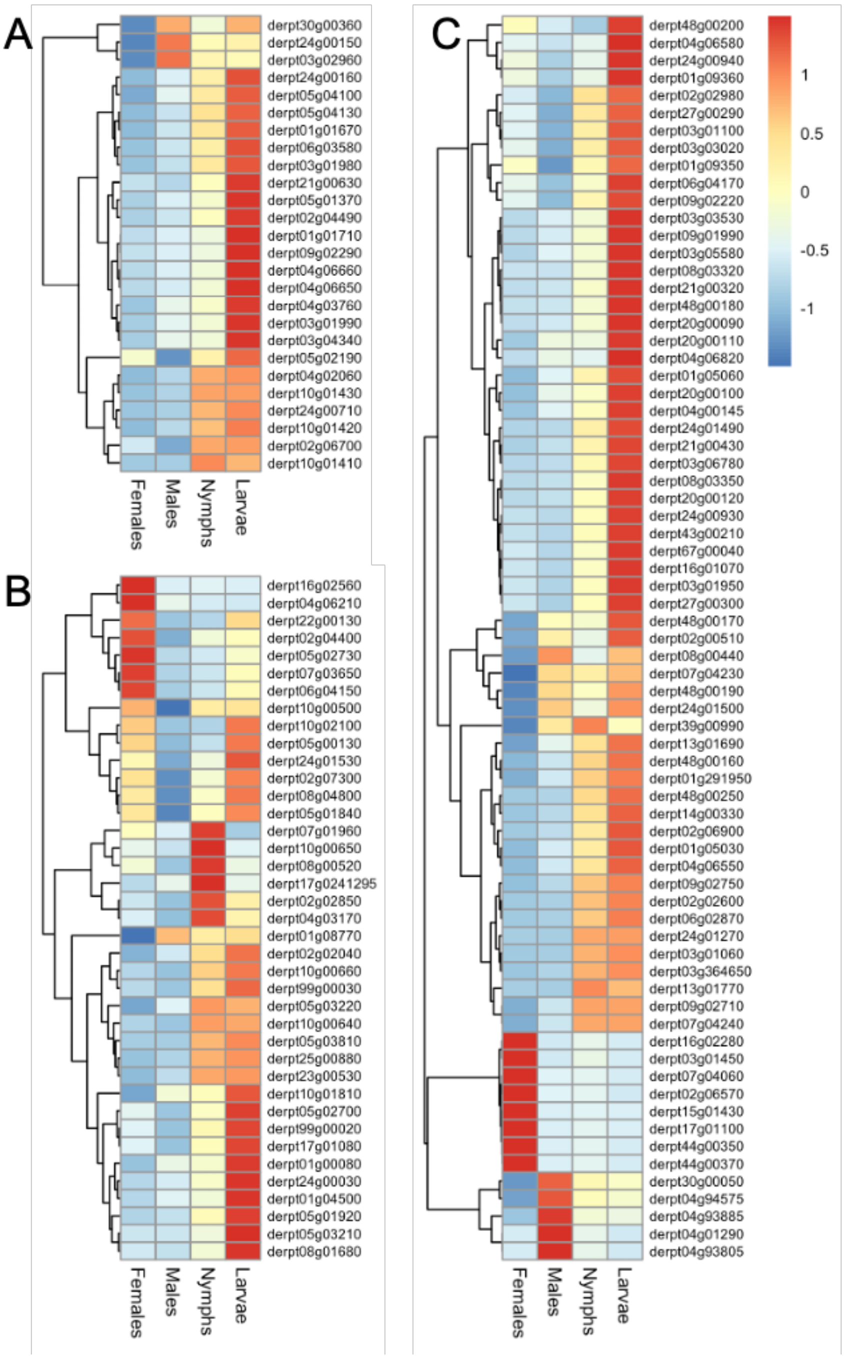

Figure S6. Stage-specific expression heatmap of all annotated peptidase genes in *D. pteronyssinus*. The darker red color indicates a higher transcript per million (TPM) average (n = 3 replicated libraries). Boxes next to the gene clustering dendrogram indicate known allergen genes; colored boxes contiguous to them shown peptidase families according Enzyme Commission numbers (EC nr); as indicated in both legends, respectively. Gene IDs are depicted in each plot. Numbers located at branch roots indicate different gene clusters.

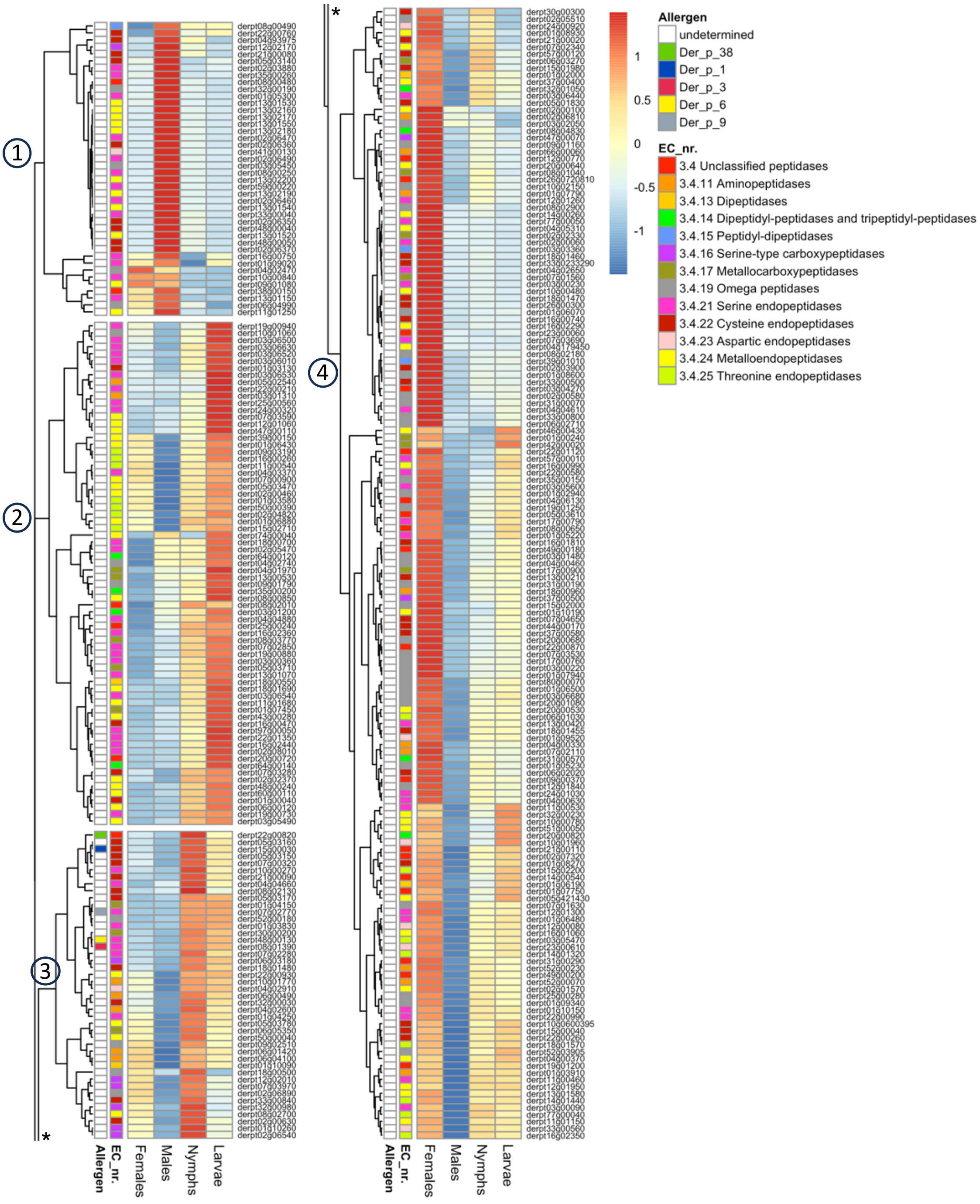

Figure S7. Stage-specific expression heatmap of potential horizontally transferred genes in *D. pteronyssinus*. The darker red color indicates a higher transcript per million (TPM) average (n = 3 replicated libraries). Colored boxes next to the gene clustering dendrogram indicate the functional category of each gene, as indicated in the legend. Allergen-related names are indicated in each plot together with gene ID (in brackets). Gene IDs are depicted in each plot. Numbers located at branch roots indicate different gene clusters.

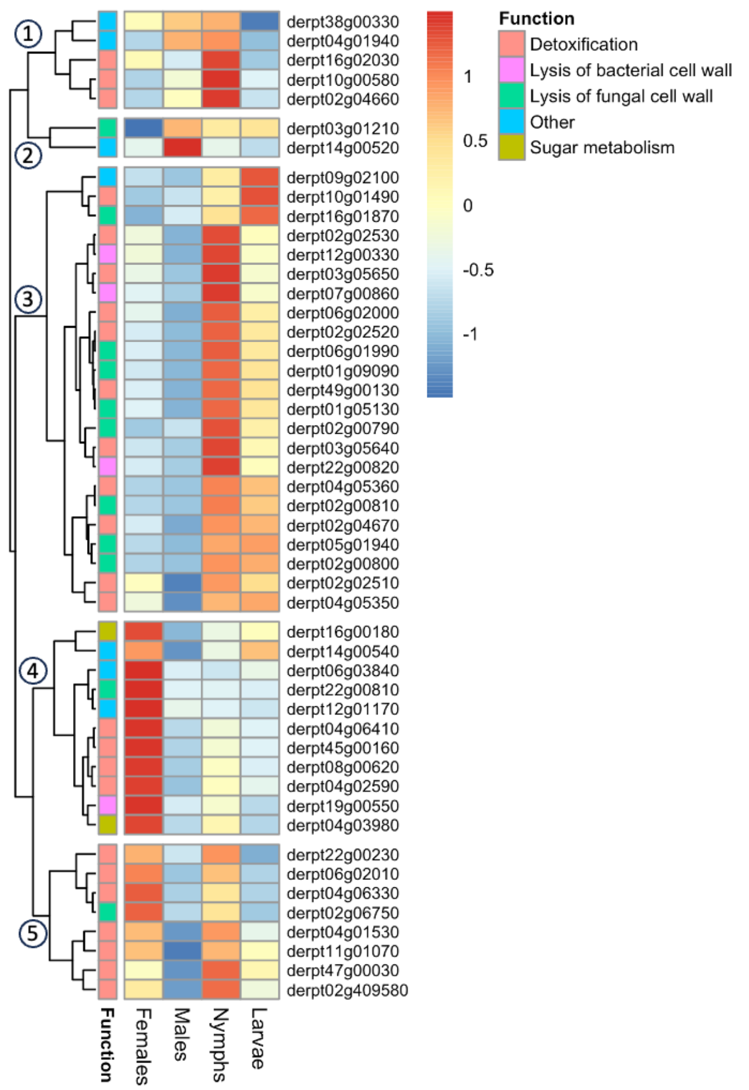

SUPPLEMENTARY TABLES:

Table S1. RNA sequencing data of stage-specific *D. pteronyssinus* Illumina libraries.

RNAseq libraries [1]	Average total reads/library	Alignment rate to *Dp* assembly (%) [2]
**Females (n=3)**	24,313,936	76.1 ± 0.6
**Males (n=3)**	25,108,996	51.3 ± 6.0
**Nymphs (n=3)**	24,677,562	73.2 ± 3.9
**Larvae (n=3)**	22,281,857	67.0 ± 3.5

[1] RNAseq libraries were obtained using total RNA extracted from purified mite body stages (3 replicates each)

[2] "Alignment rate" denotes the percentage of RNAseq reads being mapped to *D. pteronyssinus* (*Dp*) assembly GCA_027571235.1 after HISAT2 alignment. Figures are averages ± SEM.

Table S2. Differential expression of selected genes estimated by RT-qPCR

		**Females vs Larvae** (positive fold-change values indicate overexpression in Females)			**Females vs Nymphs** (positive fold-change values indicate overexpression in Females)			**Females vs Males** (positive fold-change values indicate overexpression in Females)			**Males vs Larvae** (positive fold-change values indicate overexpression in Males)			**Males vs Nymphs** (positive fold-change values indicate overexpression in Males)			**Nymphs vs Larvae** (positive fold-change values indicate overexpression in Nymphs)		
		**RT-qPCR**		**RNAseq**	**RT-qPCR**		**RNAseq**	**RT-qPCR**		**RNAseq**	**RT-qPCR**		**RNAseq**	**RT-qPCR**		**RNAseq**	**RT-qPCR**		**RNAseq**
**Locus ID [1]**	**Description**	**FC [2]**	***P* value**	**FC [3]**	**FC [2]**	***P* value**	**FC [3]**	**FC [2]**	***P* value**	**FC [3]**	**FC [2]**	***P* value**	**FC [3]**	**FC [2]**	***P* value**	**FC [3]**	**FC [2]**	***P* value**	**FC [3]**
derpt15g00030*	Peptidase C1A; Cathepsin L-like; Der p 1 allergen	**-1,61**	**0,0044**	-1,82	**-2,22**	**0,0005**	-2,67	**-1,48**	**0,021**	ns	-1,09	0,421	-1,56	**-1,50**	**0,0124**	-2,28	**1,38**	**0,0089**	1,46
derpt06g02040*	ML domain protein MLDP-1; Der p 2 allergen	1,14	0,2817	ns	**-1,43**	**0,0196**	-1,66	1,05	0,6935	ns	1,09	0,758	-1,20	-1,50	0,0836	-1,79	**1,63**	**0,0174**	1,49
derpt08g01390*	Peptidase S1A; trypsin-like; Der p 3 allergen	**-1,69**	**0,0002**	-2,14	**-2,63**	**<0,0001**	-2,54	**-1,54**	**0,0163**	-1,43	-1,10	0,3821	-1,50	**-1,71**	**0,0082**	-1,78	**1,55**	**0,0012**	ns
derpt02g04590*	Alpha-amylase; Der p 4 allergen	**-2,08**	**0,0056**	-1,71	**-2,80**	**0,0014**	-3,02	**-3,02**	**0,0251**	-1,49	1,45	0,3671	ns	1,08	0,9804	-2,03	1,35	0,1022	1,77
derpt08g03410*	Der p 5 allergen	-1,13	0,4075	-1,41	**-2,74**	**0,0001**	-1,99	1,11	0,5142	ns	-1,25	0,3224	-1,40	**-3,05**	**0,0042**	-1,97	**2,44**	**0,0018**	1,41
derpt24g00220*	Bactericidal permeability-increasing like protein Grp7-1; Der p 7 allergen	-1,36	0,0849	-1,40	**-2,09**	**0,0055**	-1,90	-2,39	0,0661	-1,44	1,76	0,2036	ns	1,14	0,871	-1,32	**1,54**	**0,0457**	1,36
derpt24g00270*	Tropomyosin; Der p 10 allergen	**-4,82**	**0,001**	-3,56	**-3,42**	**<0,0001**	-3,46	**-6,36**	**0,0005**	-4,46	1,32	0,3229	ns	**1,86**	**0,0307**	ns	-1,41	0,1631	ns
derpt08g03400*	Der p 21 allergen	**-1,75**	**0,022**	-1,46	**-3,92**	**0,0002**	-2,27	1,07	0,7003	ns	**-1,87**	**0,0325**	-1,58	**-4,17**	**0,001**	-2,45	**2,24**	**0,003**	1,56
derpt33g0233290	Peptidase C1A homologue, Cathepsin L-like	**9,81**	**0,001**	12,07	**4,37**	**0,0005**	5,50	**3,73**	**0,0103**	5,20	2,63	0,0561	2,32	1,17	0,7686	ns	**2,24**	**0,0225**	**2,20**
derpt02g06280	Ubiquitin-like domain superfamily	**-1,31**	**0,0416**	ns	-1,03	0,5901	ns	**-2,47**	**0,0082**	-1,57	1,89	0,0607	1,95	**2,40**	**0,0115**	1,81	-1,27	0,0824	ns
derpt06g01930	Ferritin-like protein FERR-2; Der p 30 allergen-like	**1,45**	**0,0283**	1,59	**-1,31**	**0,0428**	-1,46	-1,24	0,1388	ns	**1,79**	**0,0091**	1,69	-1,06	0,5738	-1,37	**1,90**	**0,0041**	**2,32**
derpt03g02900*	Actin family protein	**-1,80**	**0,0016**	-4,62	**-1,88**	**0,0011**	-4,15	-2,81	0,052	-4,42	1,56	0,5259	ns	1,50	0,5943	ns	1,04	0,6309	ns

[1] An asterisk indicates genes for which raw RT-qPCR data (Ct values) were generated in a previous study [9] and re-processed for this work. The rest of genes were analyzed *de novo*

[2] Transcription fold-change (FC) as assessed by reverse transcription and real time quantitative PCR (RT-qPCR) for each pairwise comparison between sexes/developmental stages; positive fold-change values indicate overexpression in the first sex/developmental stage under comparison, negative values indicate underexpression in this same sex/stage. RT-qPCR was performed following previously described methods [9]. Statistical differences between Cq' values (log2 transformation of normalized relative quantities) were assessed by unpaired t-test; *P* values ≤ 0.05 are highlighted in bold digits. Primer pairs for the studied gene set and reference genes used for normalization (RPL13a, RPS18, a-Tubulin) are shown in Table S3

[3] Transcription fold-change (FC) as assessed by RNAseq for each pairwise comparison between sexes/developmental stages; positive fold-change values indicate overexpression in the first sex/developmental stage under comparison, negative values indicate underexpression in this same sex/stage. Values are indicated only when adjusted *P* values are ≤ 0.05 (see additional file 2); "ns" denotes "not significant differences"

Table S3. List of qPCR primers

Gene [1]	Locus ID	Description	qPCR primers (5'-3')	Efficiency (%)
Der p 1 [a]	derpt15g00030	Peptidase C1A; Cathepsin L-like; Der p 1 allergen	F: GCCGTCATTATTGGCATCAAAG<br>R: TCGACACCTTGTGCGTTAC	93.77
Der p 2 [a]	derpt06g02040	ML domain protein MLDP-1; Der p 2 allergen	F: ACCAGGATGCCATGGTTCAG<br>R: ACCATCGATTGAGGCTTTGATT	95.11
Der p 3 [a]	derpt08g01390	Peptidase S1A; trypsin-like; Der p 3 allergen	F: TGATTCTGGTGGACCGGTTG<br>R: CACCTGGATAACCTTTACGTGC	92.31
Der p 4 [a]	derpt02g04590	Alpha-amylase; Der p 4 allergen	F: GGGCCGCATTATTGAATTCCG<br>R: TCACCACTGGGAACCAATCC	90.15
Der p 5 [a]	derpt08g03410	Der p 5 allergen	F: ACGTGGTGTACTTGATCGTCT<br>R: TCACCACTGGGAACCAATCC	90.84
Der p 7 [a]	derpt24g00220	Bactericidal permeability-increasing like protein Grp7-1; Der p 7 allergen	F: CCGCAGCTTTTGTTGCCG<br>R: ATTGCAGCGACGGCTTCATC	94.02
Der p 10 [a]	derpt24g00270	Tropomyosin; Der p 10 allergen	F: ACGTGCCGAAACTGGTGAAT<br>R: TGCTTCTTCACGTTGTTGTGC	95.68
Der p 21 [a]	derpt08g03400	Der p 21 allergen	F: ACCCTTTTCGCTGCCATTGT<br>R: ACGATCGAATGCCATACGCC	90.70
Der p 30-iso [b]	derpt06g01930	Ferritin-like protein FERR-2; Der p 30 allergen-like	F: AGAAGAACGTGAACATGCTGAA<br>R: TGACCAATCTTGTTGTACGGGT	91.18
DpPap-EH [c]	derpt33g0233290	Peptidase C1A homologue, Cathepsin L-like	F: GGGCATTTGCAACTATAGCATC<br>R: TCACCTCTACAACCATGTTTGG	91.43
Ubiquitin [c]	derpt02g06280	Ubiquitin-like domain superfamily	F: GGGATTGATCTTTGCCGGAAAG<br>R: CGTTTTACCAGTTAGGGTCTTG	92.96
Actin [a]	derpt03g02900	Actin family protein	F: GCCGGTGATGATGCTCCA<br>R: ATCTTTGTTGCCCATACCAACC	101.18
RPL13a [a]	derpt06g01480	Ribosomal protein L13, eukaryotic/archaeal	F: AACGTGGTGATTTGGCACTC<br>R: GTAATGCAGCCGGTACAACC	97.17
a-Tubulin [a]	derpt06g01480	Alpha-tubulin ATUB-1; Der p 33 allergen	F: TCAATCACTGCTTCGCTTCG<br>R: CCGAAATAACCGGTGCATAGG	94.66
RPS18 [c]	derpt02g04130	Ribosomal protein S13-like, H2TH	F: CGACAGAAAGATATTCGTGATGG<br>R: TCGAAGACCACGATGAGCG	94.62

[1] First published in: a) Vidal-Quist *et al.* (2015) [9]; b) Vidal-Quist *et al.* (2024) [4]; c) this study

Table S4. List of genes encoding cuticular proteins of previously-described families.

Gene ID	CP Family [1]	CutProtFam-Pred [2]	Nr. CBD [3]
derpt05g03220	CPAP	CPAP1	1
derpt24g00030	CPAP	CPAP1	1
derpt05g01920	CPAP	CPAP1	1
derpt05g03810	CPAP	CPAP1	1
derpt05g00130	CPAP	CPAP1	1
derpt06g04150	CPAP	CPAP1	1
derpt05g03210	CPAP	CPAP1	1
derpt07g03650	CPAP	CPAP1	1
derpt05g02700	CPAP	CPAP1	1
derpt16g02560	CPAP		1
derpt02g02040	CPAP		1
derpt10g01810	CPAP		1
derpt10g00650	CPAP		1
derpt10g00660	CPAP		1
derpt17g0241295	CPAP		1
derpt99g00020	CPAP		1
derpt07g01960	CPAP		1
derpt10g00500	CPAP		1
derpt02g04400	CPAP		1
derpt22g00130	CPAP		1
derpt99g00030	CPAP		1
derpt17g01080	CPAP		1
derpt24g01530	CPAP		1
derpt08g04800	CPAP		1
derpt04g06210	CPAP		1
derpt05g02730	CPAP		1
derpt23g00530	CPAP	CPAP3	2
derpt25g00880	CPAP		2
derpt02g02850	CPAP		2
derpt05g01840	CPAP		2
derpt10g02100	CPAP		2
derpt08g00520	CPAP		2
derpt01g00080	CPAP	CPAP3	3
derpt08g01680	CPAP	CPAP3	3
derpt01g04500	CPAP	CPAP3	3
derpt02g07300	CPAP	CPAP3	3
derpt04g03170	CPAP		3
derpt01g08770	CPAP		4
derpt10g00640	CPAP		7
derpt04g03760	CPR	CPR_RR-1	n.d.
derpt30g00360	CPR	CPR_RR-2	n.d.
derpt01g01670	CPR	CPR_RR-2	n.d.
derpt05g04130	CPR	CPR_RR-2	n.d.
derpt10g01420	CPR	CPR_RR-2	n.d.
derpt03g04340	CPR	CPR_RR-2	n.d.
derpt03g02960	CPR	CPR_RR-2	n.d.
derpt24g00150	CPR	CPR_RR-2	n.d.
derpt04g06660	CPR	CPR_RR-2	n.d.

derpt05g01370	CPR	CPR_RR-2	n.d.
derpt05g04100	CPR	CPR_RR-2	n.d.
derpt04g06650	CPR	CPR_RR-2	n.d.
derpt01g01710	CPR	CPR_RR-2	n.d.
derpt03g01990	CPR	CPR_RR-2	n.d.
derpt24g00160	CPR	CPR_RR-2	n.d.
derpt03g01980	CPR	CPR_RR-2	n.d.
derpt09g02290	CPR	CPR_RR-2	n.d.
derpt24g00710	CPR	CPR_RR-2	n.d.
derpt02g04490	CPR	CPR_RR-2	n.d.
derpt10g01410	CPR	CPR_RR-2	n.d.
derpt10g01430	CPR	CPR_RR-2	n.d.
derpt06g03580	CPR	CPR_RR-2	n.d.
derpt02g06700	CPR	CPR_RR-2	n.d.
derpt21g00630	CPR	CPR_RR-2	n.d.
derpt05g02190	CPR	CPR_RR-2	n.d.
derpt04g02060	CPR		n.d.
derpt04g06580	CPLCP		n.d.

[1] Cuticular protein (CP), cuticular protein analogous to peritrophin (CPAP), cuticular protein with chitin-binding Rebers and Riddiford (R&R) consensus (CPR), cuticular protein of low complexity and proline-rich (CPLCP)

[2] Cuticular protein family prediction as per the CutProtFam-Pred webserver [2]

[3] Number of PF01607-like chitin-binding domains (CBD) based on InterPro domain and manual annotations; n.d. denotes “not detected”

Table S5. List of genes encoding hypothetical cuticular proteins (CPH) of undescribed families.

Gene ID	Length (aa)	Homology Group	Non-polar aa (%)	Gly %	Pro %	Ala %	Nr. Cys (excluding SP)	AAP(A/V/L) motif (nr.)	Gly-dense motifs	PV/PY-rich	Tandem repeats
derpt02g02600	475	1	66.5	10.5	7	20.3	0	15	Y		
derpt13g01770	487	1	58	12.8	5.1	8.6	0		Y		
derpt48g00250	170	1	53.8	1.8	8.9	7.7	0				
derpt24g01270	287	1	55.2	7	7	9.8	0		Y		
derpt09g01990	302	1	47.5	0.7	10	3.7	0				
derpt24g01490	218	1	67.3	30.9	3.2	4.1	0		Y		
derpt48g00200	174	1	63	20.2	4.6	3.5	0		Y		
derpt03g05580	286	1	59.3	31.9	2.8	2.1	0		Y		
derpt48g00170	219	1	58.3	21.6	3.7	3.2	0		Y		
derpt48g00190	184	1	62.3	23.5	4.9	2.7	0		Y		
derpt20g00100	222	1	62.4	29.9	3.2	6.3	0		Y		
derpt06g02870	286	1	47.4	3.5	10.2	8.1	0				
derpt24g01500	387	1	69.2	6	9.3	26.4	0	11			
derpt48g00180	237	1	62.7	17.8	4.7	11	0		Y		
derpt48g00160	331	1	77.9	23.3	6.1	16.7	0		Y		
derpt20g00090	266	1	66.4	33.6	2.6	7.5	0		Y		
derpt24g00940	313	1	50	9.6	6.4	5.4	0		Y		
derpt08g03320	321	1	75.3	46.6	3.1	2.5	0		Y		
derpt08g00440	632	1	56.4	32	3.3	4.1	0		Y		
derpt21g00430	308	1	50.8	3.9	9.4	8.1	0				
derpt20g00110	224	1	58.7	22	3.1	8.5	0		Y		
derpt03g03530	242	1	68.9	34.9	3.7	3.7	0		Y		
derpt03g01060	223	1	55.4	6.3	8.1	12.2	0		Y		
derpt08g03350	199	1	49	6.6	5.1	9.6	0				
derpt24g00930	403	1	42	6.7	5.2	4	0		Y		
derpt03g01950	189	1	53.7	16	4.8	6.4	0		Y		
derpt06g04170	181	1	53.9	13.3	7.2	4.4	0		Y		
derpt03g06780	235	1	73.1	35	5.1	4.7	0		Y		
derpt67g00040	238	1	73.4	35.9	5.1	4.6	0		Y		
derpt20g00120	286	1	58.2	25.3	3.5	5.6	0		Y		
derpt03g01100	305	1	51.3	15.5	5.3	6.3	0		Y		
derpt03g03020	480	1	47	5.8	8.1	7.1	0		Y		
derpt04g01290	142	2	56.7	0	24.1	5.7	4				
derpt04g93805	97	2	66.7	4.2	29.2	6.3	2				
derpt27g00290	131	3	71.5	13.1	13.1	6.2	0		Y		
derpt27g00300	122	3	75.2	14.1	14.1	8.3	0		Y		
derpt04g00145	128	4	66.1	8.7	11.8	2.4	0		Y	Y	
derpt04g06820	130	4	72.9	10.1	11.6	4.7	0		Y	Y	
derpt21g00320	268	4	70	29.6	6.4	1.9	0		Y	Y	
derpt01g05060	112	5	80.2	11.7	9.9	23.4	0	2			
derpt01g05030	150	5	77.9	9.4	16.1	16.8	0			Y	
derpt02g00510	81	6	87.5	58.8	0	3.8	0		Y		
derpt04g93885	60	6	69.5	23.7	1.7	5.1	0		Y		
derpt03g364650	89	6	60.2	23.9	0	1.1	0		Y		
derpt39g00990	65	6	76.6	37.5	0	4.7	0		Y		
derpt09g02710	99	6	88.8	31.6	6.1	16.3	0		Y		
derpt07g04230	73	6	81.9	37.5	0	6.9	0		Y		

derpt07g04240	66	6	75.4	29.2	1.5	6.2	0		Y		
derpt09g02750	99	6	91.8	42.9	1	11.2	0		Y		
derpt13g01690	80	6	68.4	40.5	0	5.1	0		Y		
derpt16g01070	111	6	79.1	36.4	2.7	1.8	1		Y		
derpt43g00210	100	6	75.8	22.2	12.1	8.1	0		Y		
derpt44g00350	514	7	45.8	13.3	13.8	1.6	1		Y		Y
derpt44g00370	261	7	61.2	8.1	16.5	1.9	0		Y		
derpt16g02280	255	8	87.8	2.4	36.6	30.3	2				Y
derpt03g01450	784	8	59.3	2.4	20.6	8.9	16				Y
derpt04g06550	159		79.1	8.9	15.2	17.7	0	3		Y	
derpt30g00050	103		55.9	22.5	4.9	1	0		Y		
derpt07g04060	175		80.5	29.3	9.8	7.5	1		Y		Y
derpt17g01100	174		68.2	3.5	19.1	4	0				
derpt04g94575	68		68.7	11.9	6	4.5	0				
derpt02g06570	328		75.2	2.1	23.2	20.8	1	10			Y
derpt15g01430	90		71.9	23.6	5.6	0	0		Y		
derpt14g00330	385		39.6	6.5	12.8	0.8	0		Y		
derpt02g06900	235		75.6	27.8	0	29.1	0		Y		
derpt01g09350	394		55.5	5.1	17.6	4.1	0				
derpt09g02220	491		49.8	4.9	12.4	4.7	0		Y		
derpt01g09360	414		72.9	24.9	16	6.1	0		Y		
derpt02g02980	412		44.3	4.6	12.4	4.9	0				
derpt01g291950	271		59.6	2.2	18.5	4.4	0			Y	

Acronyms: amino acids (aa); Gly (glycine); Pro or P (proline); Ala or A (alanine); Cys (cysteine); signal peptide (SP); V (valine); L (leucine); Y (tyrosine); Y (yes)

Table S6. Putative horizontally transferred genes in *D. pteronyssinus*

Gene ID	Description	Functional category	Intron [1] presence	*h*-index (donor; top bitscore) [2]	Reference
derpt07g00860	Endolytic peptidoglycan transglycosylase	Lysis of bacterial cell wall	Y	0 (Bacteria; 97)	[3]
derpt12g00330	Lysozyme	Lysis of bacterial cell wall	Y	10 (Fungi; 169)	[4]
derpt22g00820	NLPC/P60 endopeptidase; Der p 38	Lysis of bacterial cell wall	Y	-71 (Fungi; 110)	[5]
derpt01g05130	Chitosanase	Lysis of fungal cell wall	Y	253 (Bacteria; 253)	[3]
derpt02g06750	Chitosanase	Lysis of fungal cell wall	Y	219 (Bacteria; 219)	[3]
derpt01g09090	Chitinase GH18	Lysis of fungal cell wall	Y	22 (Bacteria; 341)	[3]
derpt05g01940	Chitinase GH18	Lysis of fungal cell wall	Y	-34 (Bacteria; 320)	[3]
derpt16g01870	Chitinase GH19	Lysis of fungal cell wall	Y	-1 (Fungi; 171)	[4]
derpt02g00790	Beta-1,3 glucanase	Lysis of fungal cell wall	N	-115 (Bacteria; 218)	[6]
derpt02g00800	Beta-1,3 glucanase	Lysis of fungal cell wall	N	-162 (Bacteria; 238)	[6]
derpt02g00810	Beta-1,3 glucanase	Lysis of fungal cell wall	N	-133 (Bacteria; 239)	[6]
derpt03g01210	Beta-1,3 glucanase	Lysis of fungal cell wall	Y	18 (Bacteria; 108)	[3]
derpt06g01990	Beta-1,3 glucanase	Lysis of fungal cell wall	N	-85 (Bacteria; 187)	[3]
derpt22g00810	Beta-1,3 glucanase	Lysis of fungal cell wall	Y	-3 (Bacteria; 92)	[3]
derpt08g00620	Intradiol ring-cleavage dioxygenase	Detoxification	Y	9 (Bacteria; 181)	[7]
derpt10g01490	Nitroreductase	Detoxification	Y	178 (Bacteria; 178)	This study
derpt19g00550	Trans-aconitate 2-methyltransferase	Detoxification	Y	8 (Bacteria; 82)	[3]
derpt02g02510	UGT (UDP glucuronosyltransferase)	Detoxification	Y	-106 (Bacteria; 278)	[3]
derpt02g02520	UGT	Detoxification	Y	-137 (Bacteria; 306)	[3]
derpt02g02530	UGT	Detoxification	Y	-88 (Bacteria; 301)	[3]
derpt02g04660	UGT	Detoxification	Y	-96 (Bacteria; 280)	[3]
derpt02g04670	UGT	Detoxification	Y	-81 (Bacteria; 308)	[3]
derpt03g05640	UGT	Detoxification	Y	-73 (Bacteria; 285)	[3]
derpt03g05650	UGT	Detoxification	Y	-96 (Bacteria; 305)	[3]
derpt04g01530	UGT	Detoxification	Y	-61 (Bacteria; 276)	[3]
derpt04g02590	UGT	Detoxification	Y	-105 (Bacteria; 243)	[3]
derpt04g05350	UGT	Detoxification	N	-124 (Bacteria; 270)	[3]
derpt04g05360	UGT	Detoxification	Y	-147 (Bacteria; 263)	[3]
derpt04g06330	UGT	Detoxification	N	-66 (Bacteria; 241)	[3]
derpt04g06410	UGT	Detoxification	Y	-84 (Bacteria; 248)	[3]
derpt06g02000	UGT	Detoxification	Y	-235 (Bacteria; 312)	[3]
derpt06g02010	UGT	Detoxification	Y	-194 (Bacteria; 295)	[3]
derpt10g00580	UGT	Detoxification	N	-9 (Bacteria; 294)	[3]
derpt11g01070	UGT	Detoxification	N	-54 (Bacteria; 213)	[3]
derpt16g02030	UGT	Detoxification	N	-93 (Bacteria; 292)	[3]
derpt22g00230	UGT	Detoxification	Y	-99 (Bacteria; 279)	[3]
derpt45g00160	UGT	Detoxification	N	-52 (Bacteria; 268)	[3]
derpt47g00030	UGT	Detoxification	Y	-126 (Bacteria; 287)	[3]

derpt49g00130	UGT	Detoxification	Y	-12 (Bacteria; 305)	[3]
derpt04g03980	Aldose 1-epimerase	Sugar metabolism	N	39 (Fungi; 281)	This study
derpt16g00180	Glucokinase	Sugar metabolism	Y	36 (Bacteria; 161)	This study
derpt04g01940	Asparagine synthase	Amino acid biosynthesis	Y	164 (Fungi; 751)	This study
derpt38g00330	Ribonucleoside hydrolase	Nucleic acid degradation	N	131 (Bacteria; 248)	This study
derpt14g00540	Glutaminyl-peptide cyclotransferase	Protein modification	Y	34 (Fungi; 162)	This study
derpt12g01170	DNA photolyase class 1	UV damage DNA repair	N	39 (Bacteria; 332)	This study
derpt09g02100	Stress response protein	Bacterial stress response	N	126 (Bacteria; 126)	[3]
derpt06g03840	RNA 2'-phosphotransferase	RNA splicing	N	12 (Bacteria; 82)	[3]
derpt14g00520	Terpene synthase	Biosynthesis of terpenes	Y	9 (Bacteria; 84)	[3]

[1] Introns were scored manually from visualization in the genome browser GenomeView, available at ORCAE [8]. "Y" denotes "yes"; "N" denotes "no".

[2] *h*-index as computed in our study by subtracting the best BLASTp bitscore of a non-Acari invertebrate protein (as retrieved from RefSeq NCBI Reference Sequence Database after excluding mite and tick proteins from metazoan invertebrate proteins) from the best BLASTp bitscore of a bacterial or fungal protein (as retrieved from RefSeq Fungi or Bacteria databases, respectively). The taxonomic kingdom (Bacteria or Fungi) of the top BLASTp bitscore, as potential gene donor phylum, is indicated in brackets together with its actual bitscore against the *Dp* protein under study.

Additional file 1 references:

1. KEGG: Kyoto Encyclopedia of Genes and Genomes. https://www.kegg.jp. Accessed 21 Feb 2025.
2. CutProtFam - Pred. A Cuticular Protein Family Prediction Tool. http://aias.biol.uoa.gr/CutProtFam-Pred/home.php. Accessed 21 Feb 2025.
3. Xiong Q, Wan AT-Y, Liu X, Fung CS-H, Xiao X, Malainual N, et al. Comparative genomics reveals insights into the divergent evolution of astigmatic mites and household pest adaptations. Mol Biol Evol. 2022;39:msac097.
4. Vidal-Quist JC, Ortego F, Rombauts S, Hernández-Crespo P. The genome-wide response of *Dermatophagoides pteronyssinus* to cystatin A, a peptidase inhibitor from human skin, sheds light on its digestive physiology and allergenicity. Insect Mol Biol. 2024;33:662–77.
5. Tang VH, Stewart GA, Chang BJ. House dust mites possess a polymorphic, single domain putative peptidoglycan d,l endopeptidase belonging to the NlpC/P60 Superfamily. FEBS Open Bio. 2015;5:813–23.
6. Waldron R, McGowan J, Gordon N, Mitchell EB, Fitzpatrick DA, Doyle S. Characterisation of three novel β-1,3 glucanases from the medically important house dust mite *Dermatophagoides pteronyssinus* (airmid). Insect Biochem Mol Biol. 2019;115:103242.
7. Njiru C, Xue W, De Rouck S, Alba JM, Kant MR, Chruszcz M, et al. Intradiol ring cleavage dioxygenases from herbivorous spider mites as a new detoxification enzyme family in animals. BMC Biol. 2022;20:131.
8. Online Resource for Community Annotation of Eukaryotes. Dermatophagoides pteronyssinus. https://bioinformatics.psb.ugent.be/orcae/overview/Derpt_pub. Accessed 21 Feb 2025.
9. Vidal-Quist JC, Ortego F, Lombardero M, Castañera P, Hernández-Crespo P. Allergen expression in the European house dust mite Dermatophagoides pteronyssinus throughout development and response to environmental conditions. Med Vet Entomol. 2015;29:137–46.
